# Effect of short term diet restriction on gene expression in the bovine hypothalamus using next generation RNA sequencing technology

**DOI:** 10.1186/s12864-017-4265-6

**Published:** 2017-11-09

**Authors:** Daragh Matthews, Michael G. Diskin, David A. Kenny, Christopher J. Creevey, Kate Keogh, Sinead M. Waters

**Affiliations:** 1 Animal and Grassland Research and Innovation Centre, Teagasc, Mellows Campus, Athenry, Co. Galway Ireland; 2 School of Agriculture and Food Science, University College Dublin, Belfield, Dublin 4 Ireland; 3 Animal and Bioscience Research Department, Animal and Grassland Research and Innovation Centre, Teagasc, Grange, Dunsany, Co. Meath Ireland

**Keywords:** mRNAseq, Bovine, Hypothalamus, Energy homeostasis, Reproduction, Dietary restriction

## Abstract

**Background:**

Negative energy balance (NEB) is an imbalance between energy intake and energy requirements for lactation and body maintenance affecting high-yielding dairy cows and is of considerable economic importance due to its negative impact on fertility and health in dairy herds. It is anticipated that the cow hypothalamus experiences extensive biochemical changes during the early post partum period in an effort to re-establish metabolic homeostasis. However, there is variation in the tolerance to NEB between individual cows. In order to understand the genomic regulation of ovulation in hypothalamic tissue during NEB, mRNA transcriptional patterns between tolerant and sensitive animals were examined. A short term dietary restriction heifer model was developed which induced abrupt onset of anoestrus in some animals (Restricted Anovulatory; RA) while others maintained oestrous cyclicity (Restricted Ovulatory; RO). A third control group (C) received a higher level of normal feeding.

**Results:**

A total of 15,295 genes were expressed in hypothalamic tissue. Between RA and C groups 137 genes were differentially expressed, whereas between RO and C, 32 genes were differentially expressed. Differentially expressed genes were involved in the immune response and cellular motility in RA and RO groups, respectively, compared to C group. The largest difference between groups was observed in the comparison between RA and RO heifers, with 1094 genes shown to be significantly differentially expressed (SDE). Pathway analysis showed that these SDE genes were associated with 6 canonical pathways (*P* < 0.01), of which neuroactive ligand-receptor interaction was the most significant. Within the comparisons the main over-represented pathway functions were immune response including neuroprotection (*CXCL10*, *Q1KLR3*, *IFIH1*, *IL1* and *IL8;* RA v C and RA v RO); energy homeostasis (*AgRP* and *NPY*; RA v RO); cell motility (*CADH1, DSP* and *TSP4;* RO v C) and prevention of GnRH release (*NTSR1 IL1α*, *IL1β*, *NPY* and *PACA*; RA v RO).

**Conclusions:**

This information will assist in understanding the genomic factors regulating the influence of diet restriction on fertility and may assist in optimising nutritional and management systems for the improvement in reproductive performance.

**Electronic supplementary material:**

The online version of this article (10.1186/s12864-017-4265-6) contains supplementary material, which is available to authorized users.

## Background

Over the decades great emphasis has been placed on selective breeding for milk yield in dairy cows resulting in cows being unable to meet the energy demands of maintenance and lactation with consequential mobilisation of body reserves to meet these demands. Such cows are described as being in NEB. Excessive and/or prolonged NEB negatively impacts on reproductive performance and increases the cows’ susceptibility to disease [1]. During periods of severe NEB, animals are forced to channel available energy toward survival and away from processes such as reproduction [2]. Normal ovarian function is therefore delayed in such animals until the energy deficit is at least partially corrected. There is also evidence that a proportion of animals will resume cyclicity sooner than others despite all animals being at a similar energy balance [3, 4]. Mackey et al. [5] reported that some animals will continue to ovulate, while others will become anovulatory when placed on a severely restricted diet despite no differences in weight or body condition score (BCS) at the beginning of a feeding phase.

Hormones such as insulin [6], IGF-1 [7], GH [8] leptin [9], and nutrients such as glucose [6] and fatty acids [9] have been implicated in signalling nutritional status. These signals act within the hypothalamus to regulate feed intake, energy expenditure and neuroendocrine functions including reproduction [10–12]. The hypothalamus is central to the neural control of homeostasis. Neurons in the hypothalamus are responsive to changes in metabolic status [12, 13] and appear to play an important role in mediating the effects of nutrition on reproduction via the hypothalamic-pituitary ovarian axis [14]. Gonadotrophin releasing hormone (GnRH) is the primary reproductive hormone secreted from the hypothalamus that integrates a multitude of internal and environmental cues to regulate the secretion of luteinizing hormone (LH) and follicle stimulating hormone (FSH) from the anterior pituitary gland [15].

The effects of nutrition on reproduction are most likely mediated through the hypothalamic-pituitary-ovarian axis [16]. We have developed a short term dietary restriction heifer model facilitating animals to be characterised as sensitive or tolerant to an energy deficit based on their ability to ovulate while fed a restricted diet [17]. Approximately a third of heifers became anoestrous due to diet restriction. As expected, follicular growth rate and maximum diameter were reduced by diet restriction, with larger dominant follicles more likely to ovulate. Walsh et al. [18] showed that dietary restriction altered gene expression in the dominant follicle of the ovary potentially leading to reduced oestradiol synthesis, FSH-responsiveness and IGF signalling in granulosa, and LH-responsiveness in theca cells of dominant follicles. Furthermore, steroid biosynthesis within developing follicles was also altered suggesting that cholesterol transport into mitochondria to initiate steroidogenesis was affected [19].

Genes expressed in the hypothalamus are suggested to be involved in processing estrous behaviour in cattle [20, 21]. While candidate gene expression studies have been performed [13, 17], there are no published studies on the application of RNAseq to analyse hypothalamic tissue of individual animals in response to diet restriction. Thus the objective of this study was to compare differences in transcriptional profiles in hypothalamic tissue between these two groups of animals in an energy deficit but with divergent reproductive performance, and to a third control group on a higher level of feeding using RNAseq and pathway analysis.

## Results

### Transcriptional profile of the bovine hypothalamus

On average close to 25.5 million fragments were sequenced for each sample. Of these 16 million, or approximately 60%, were mapped to the bovine genome by bowtie (Table 1). On average, 9 million mapped reads remained after filtering out reads with more than 1 alignment to the genome and reads that mapped to exactly the same position on the genome (i.e. putative PCR duplicates). Approximately 4 million reads per sample were mapped to annotated exons by HTseq. An overview of these data is given in Table 1. Following exclusion of genes with fewer than 5 reads for one sample in any of the 3 groups, 15,295 genes were detected as expressed. The RNAseq data have been deposited in NCBI’s Gene Expression Omnibus [22] and are accessible through GEO Series accession number GSE49540.

**Table 1:** Summary of sequencing read alignment to the bovine genome

Process	
Total sequenced fragments	25,512,814
Fragments mapped to nuclear genome	16,035,986
Percentage mapped	62.9%
Uniquely mapped fragments	12,655,723
Percentage uniquely mapped	78.9%
Fragments without duplicates	9,436,348
Fragments mapped to annotated genes	3,888,758

### Identification of significantly differentially expressed (SDE) genes

Three comparisons of differential gene expression were carried out. These were RA v C, RA v RO, and RO v C. SDE genes were called at a false discover rate (FDR) of 0.1 using the DEseq package in R, which models data as a negative binomial distribution. After statistical analysis with DEseq a total of 69 genes had increased expression and 68 displayed decreased expression in the RA relative to C comparison (Additional file 1: Table S1). Fifteen genes had increased expression and 17 had decreased expression in the RO relative to C comparison (Additional file 2: Table S2). The comparison with the greatest number of SDE genes was RA v RO where 351 genes were observed to have increased expression and 743 decreased expression in the RA relative to RO comparison (Additional file 3: Table S3).

### Pathway analysis

To gain insights into the biological processes occurring in the hypothalamus that result in anovulation due to diet restriction, three gene ontology and pathway analysis tools were applied. These included GOseq, Innatedb and Ingenuity Pathway Analysis.

#### GOseq

Enrichment of SDE genes in gene ontology (GO) terms was tested. GO annotations of genes with a FDR < 0.1 from DEseq analysis were tested against all gene annotations in the GOseq database. 145 GO terms were enriched (*P* < 0.01) in the comparison between RA and C. 47 GO terms were enriched (P < 0.01) between RO and C. And finally, 238 GO terms were enriched (P < 0.01) when RA and RO were compared. The top 10 GO terms for each comparison are listed in Table 2.

**Table 2:** Enriched GO terms for all comparisons

Go Term	Ontology^a^	*P* value
Restricted Anovulatory v Control		
Defense response	bp	4.79E-09
Type I interferon-mediated signaling pathway	bp	1.15E-06
Cellular response to type I interferon	bp	1.15E-06
Response to type I interferon	bp	1.29E-06
Response to other organism	bp	1.70E-06
Immune response	bp	3.71E-06
Response to biotic stimulus	bp	3.96E-06
Response to virus	bp	5.36E-06
Innate immune response	bp	5.47E-06
Gated channel activity	mf	8.57E-06
Restricted Ovulatory v Control		
Establishment of synaptic specificity at neuromuscular junction	bp	0.001550394
Collagen type XV	cc	0.001734458
Cell-cell adherens junction	cc	0.001885389
Cellular component disassembly at cellular level	bp	0.002858457
Cellular component disassembly	bp	0.002995423
Macrophage colony-stimulating factor receptor binding	mf	0.003064441
Endothelial cell-cell adhesion	bp	0.003122893
Thyroid-stimulating hormone receptor activity	mf	0.003136574
Myoblast migration	bp	0.003153657
Transforming growth factor beta receptor complex assembly	bp	0.003216917
Restricted Ovulatory v Restricted Anovulatory		
Neuron projection	cc	6.75E-09
Cell periphery	cc	2.54E-08
Plasma membrane	cc	3.71E-08
G-protein coupled receptor protein signaling pathway	bp	1.87E-07
Receptor activity	mf	1.18E-06
Signal transducer activity	mf	1.20E-06
Molecular transducer activity	mf	1.20E-06
Dendrite	cc	1.85E-06
Cell surface receptor linked signaling pathway	bp	7.16E-06
Intrinsic to membrane	cc	9.48E-06

^a^Gene ontology terms: bp: biological process; mf: molecular function; cc: cellular component

#### Innatedb

SDE genes were normalised for gene length bias and were then mapped to the Innatedb database for pathway analysis using GOseq. From this mapping, a total of 12, 15, and 6 genetic pathways were found to be enriched (P < 0.01) in the RA v C, RO v C, and RA v RO comparisons, respectively. Only 16 and 6 SDE genes resulted in the enrichment of these pathways in the RA v C and RO v C comparisons. However, 58 SDE genes resulted in 6 enriched pathways in the RA v RO comparison. The small number of SDE genes resulting in a number of pathways becoming enriched was due to genes being present in multiple pathways. Pathways representing each comparison are listed in Tables 3, 4 and 5.

**Table 3:** Enriched genetic pathways between Restricted Anovulatory and Control groups from Innatedb

Pathway name	Genes	Total no. of genes in pathway	P value
RIG-I-like receptor signaling pathway	*CXCL10* ↑, *Q1KLR3* ↑, *IFIH1* ↑, *IL8* ↑	70	0.000815541
Activation of NMDA receptor upon glutamate binding and postsynaptic events	*ADCY1* ↓, *GRIN2A* ↓, *GRIN2C* ↓	32	0.002998833
Post NMDA receptor activation events	*ADCY1* ↓, *GRIN2A* ↓, *GRIN2C* ↓	32	0.002998833
Long-term potentiation	*ADCY1* ↓, *GRIN2A* ↓, *GRIN2C* ↓, *ITPR1* ↓	68	0.003241851
Cytosolic DNA-sensing pathway	*CXL10* ↑, *Q1KLR3* ↑, *ZBP1* ↑	55	0.003482075
Transmission across Chemical Synapses	*ADCY1* ↓, *GRIN2A* ↓, *GRIN2C* ↓, *SLC6A3* ↓	76	0.00456686
Synaptic Transmission	*ADCY1* ↓, *GRIN2A* ↓, *GRIN2C* ↓, *SLC6A3* ↓	81	0.005631178
Gata3 participate in activating the th2 cytokine genes expression	*ADCY1* ↓, *IL1A* ↑	18	0.007137619
D-Arginine and D-ornithine metabolism	*DAO* ↓	1	0.007150141
Neuroactive ligand-receptor interaction	*CALRL* ↑, *A6QQP6* ↑, *GRIK3* ↓, *GRIN2A* ↓, *GRIN2C* ↓, *TRY2* ↑	301	0.009258012
Neuroransmitter Receptor Binding And Downstream Transmission In The Postsynaptic Cell	*ADCY1* ↓, *GRIN2A* ↓, *GRIN2C* ↓	48	0.009603978
Unblocking of NMDA receptor, glutamate binding and activation	*GRIN2A* ↓, *GRIN2C* ↓	12	0.009663882

Expression is Restricted Anovulatory relative to Control. eg. *CXL10* ↑ means RA has increased expression compared to C

**Table 4:** Enriched genetic pathways between Restricted Ovulatory and Control groups from Innatedb

Pathway name	Genes	Total no. of genes in pathway	P value
Apoptotic cleavage of cell adhesion proteins	*CADH1* ↓, *DSP* ↓	11	7.81E-05
Immunoregulatory interactions between a Lymphoid and a non-Lymphoid cell	*CADH1* ↓, *Q05B55* ↓,	74	0.001265006
Apoptotic cleavage of cellular proteins	*CADH1* ↓, *DSP* ↓	36	0.001499709
Arf6 trafficking events	*CADH1* ↓, *TSHR* ↓	39	0.001743312
Apoptotic execution phase	*CADH1* ↓, *DSP* ↓	42	0.001980774
Proteinase-activated receptor G (12/13) cascade	*PAR1* ↓	3	0.003148078
Thrombin-mediated activation of PARs	*PAR1* ↓	4	0.004671547
Platelet Activation	*PAR1* ↓, *TSP4* ↓	95	0.005884444
Signaling by GPCR	*PAR1* ↓, *TSHR* ↓	648	0.006837614
Classical antibody-mediated complement activation	*Q05B55* ↓	24	0.007641167
Proteinase-activated receptor G (q) cascade	*PAR1* ↓	6	0.007793549
Thrombin signalling G-protein cascades	*PAR1* ↓	6	0.007793549
Sumoylation as a mechanism to modulate ctbp-dependent gene responses	*CADH1* ↓	8	0.008891268
Formation of Platelet plug	*PAR1* ↓, *TSP4* ↓	114	0.00923053
Thrombin signalling through PARs	*PAR1* ↓	7	0.009310227

Expression is Restricted Ovulatory relative to Control. eg. *CADH1* ↓ means RO has decreased expression of this compared to C

**Table 5:** Enriched genetic pathways between Restricted Anovulatory and Restricted Ovulatory groups from Innatedb

Pathway name	Genes	Total no. of genes in pathway	P value
Neuroactive ligand-receptor interaction	*PACA* ↑, *AGRP* ↑, *AGTR1* ↓, *A6QL98* ↑, *A4IFF5* ↑, *CALRL* ↑, *A6QHL2* ↑, *Q9BGU4* ↓, *C6KEA7* ↑, *PAR1* ↑, *A6QQP6* ↑, *GBRR2* ↓, *GHR* ↑, *SLIB* ↑, *GPR83* ↑, *GRIK3* ↓, *GRIN2A* ↓, *GRIN2B* ↓, *GRIN2C* ↓, *GRM4* ↓, *HRH1* ↑, *5HT2A* ↑, *MC4R* ↑, *NPY* ↑, *NTSR1* ↓, *TRY2* ↑, *PF2R* ↑, *S1PR5* ↓, *TKN1* ↑, *TSHR* ↑	301	0.000211925
Toll-like receptor signaling pathway	*Q2LGB8* ↑, *CD14* ↑, *Q1JPC5* ↑, *CXL10* ↑, *CXCL9* ↑, *FADD* ↓, *IL1B* ↑, *A6QQK2* ↓, *PIK3CD* ↓, *A4IFU4* ↑, *TLR2* ↑, *TLR3* ↑, *TLR4* ↑	101	0.00365653
Caspase8 activation signalling	*Q2LGB8* ↑, *FADD* ↓	2	0.004011647
Systemic lupus erythematosus	*Q3SYT3* ↑, *C1S* ↑, *Q1JPC5* ↑, *FCGR2* ↑, *FCGR3* ↑, *GRIN2A* ↓, *GRIN2B* ↓, *Q17QG8* ↓, *RO52* ↑	95	0.004188484
Signaling by GPCR	*ADCY1* ↓, *PACA* ↑, *AGTR1* ↓, *A6QL98* ↑, *ARHGEF1* ↓, *CALRL* ↑, *A6QHL2* ↑, *CCR5* ↑, *Q9BGU4* ↓, *C6KEA7* ↑, *CXL10* ↑, *CXCL9* ↑, *PAR1* ↑, *SLIB* ↑, *A2VEA2* ↓, *GRM4* ↓, *HRH1* ↑, *5HT2A* ↑, *MC4R* ↑, *NPY* ↑, *NTSR1* ↓, *OR2W3* ↓, *PDE8B* ↑, *LOC785870* ↑, *LOC616798* ↓, *PF2R* ↑, *RGR* ↓, *S1PR5* ↓, *TKN1* ↑, *TSHR* ↑	648	0.004475761
Class A/1 (Rhodopsin-like receptors)	*AGTR1* ↓, *A6QL98* ↑, *A6QHL2* ↑, *CCR5* ↑, *CXL10* ↑, *CXCL9* ↑, *PAR1* ↑, *A2VEA2* ↓, *HRH1* ↑, *5HT2A* ↑, *MC4R* ↑, *NPY* ↑, *NTSR1* ↓, *LOC785870* ↑, *PF2R* ↑, *RGR* ↓, *S1PR5* ↓, *TKN1* ↑, *TSHR* ↑	222	0.006932285

Expression is Restricted Anovulatory relative to Restricted Ovulatory. eg. *PACA* ↑ means expression is higher in RA compared to RO

#### Ingenuity pathway analysis

To determine the molecular changes occurring that result in a proportion of heifers to become anovulatory, a 2nd gene ontology tool, Ingenuity Pathway Analysis (IPA), was used to analyse gene expression data. IPA returned a total of 9, 1 and 11 canonical pathways for RA v C, RO v C and RA v RO comparisons (Table 6).

**Table 6:** Enriched genetic pathways in all comparisons from in IPA

Pathway name	Genes	Total no. of genes in pathway	P value
Restricted Anovulatory v Control			
Activation of IRF by Cystosolic Pattern Recognition Receptors	*DDX58* ↑, *IFIH1* ↑, *IFIT2* ↑, *ZBP1* ↑	72	0.00073
Interferon Signalling	*IFIT1* ↑, *IFIT3* ↑, *MX1* ↑	36	0.00135
Role of Hypercytokinemia/hyperchemokinemia in the Pathogenesis of Influenza	*CXCL10* ↑, *IL8* ↑, *IL1A* ↑	44	0.00286
Synaptic Long Term Potentiation	*ADCY1* ↓, *GRIN2A* ↓, *GRIN2C* ↓, *ITPR1* ↓	114	0.00461
CREB Signalling in Neurons	*ADCY1* ↓, *GRIK3* ↓, *GRIN2A* ↓, *GRIN2C* ↓, *ITPR1* ↓	202	0.00515
Calcium Signalling	*CHRNA2* ↓, *GRIN2A* ↓, *GRIN2C* ↓, *ITPR1* ↓, *MYH11* ↑	207	0.00541
Glutamate Receptor Signalling	*GRIK3* ↓, *GRIN2A* ↓, *GRIN2C* ↓	69	0.00595
D-arginine and D-ornithine Metabolism	*DAO* ↓	18	0.00646
Glycosphingolipid Biosynthesis – Neolactoseries	*ST3GAL6* ↑, *ST8SIA5* ↓	64	0.00957
Restricted Ovulatory v Control			
Gα12/13 Signalling	*CDH1* ↓, *F2R* ↓	128	0.00767
Restricted Anovulatory v Restricted Ovulatory			
LXR/RXR Activation	*APOA4* ↓, *CCL2* ↑, *CD14* ↑, *IL1A* ↑, *IL1B* ↑, *MSR1* ↑, *NR1H2* ↓, *PTGS2* ↑, *RXRG* ↑, *SREBF1* ↓, *TLR3 ↑, TLR4* ↑	93	0.000344
Germ Cell-Sertoli Cell Junction Signaling	*A2M* ↑, *ACTG2* ↑, *AXIN1* ↓, *BCAR1* ↓, *EPN1* ↓, *EPN2* ↓, *GSN* ↓, *IQGAP1* ↑, *JUP* ↓, *LAMC3* ↓, *MAP3K9* ↓, *MAP3K11* ↓, *PIK3CD* ↓, *PVRL2* ↓, *RHOT2* ↓, *RND3* ↑, *TUBA8* ↓, *ZYX* ↓	167	0.000870
TREM1 Signaling	*CASP5* ↑, *CCL2* ↑, *CD86* ↑, *FCGR2B* ↑, *ICAM1* ↑, *IL1B* ↑, *TLR2* ↑, *TLR3* ↑, *TLR4* ↑	66	0.00125
Neuropathic Pain Signaling In Dorsal Horn Neurons	*GRIN2A* ↓, *GRIN2B* ↓, *GRIN2C* ↓, *GRM4* ↓, *KCNH2* ↓, *KCNN1* ↓, *KCNQ2* ↓, *KCNQ3* ↓, *PIK3CD* ↓, *PLCH2* ↓, *PRKCG* ↓, *PRKCH* ↓, *TAC1* ↑	108	0.00133
Hepatic Fibrosis / Hepatic Stellate Cell Activation	*A2M* ↑, *AGTR1* ↓, *CCL2* ↑, *CCR5* ↑, *CD14* ↑, *CXCL9* ↑, *FLT1* ↑, *ICAM1* ↑, *IGFBP5* ↓, *IL1A* ↑, *IL1B* ↑, *KDR* ↑, *MYH11* ↑, *MYH14* ↓, *STAT1* ↑, *TLR4* ↑	147	0.00159
Reelin Signalling in Neurons	*APBB1* ↓, *ARHGEF1* ↓, *ARHGEF10* ↓, *ARHGEF16* ↓, *CDK5R1* ↓, *MAP3K9* ↓, *MAP3K11* ↓, *MAPK8IP1* ↓, *MAPK8IP3* ↓, *PIK3CD* ↓, *RELN* ↑	82	0.00175
G-Protein Coupled Receptor Signalling	*ADCY1* ↓, *AGTR1* ↓, *APLNR* ↑, *C3AR1* ↑, *CALCRL* ↑, *CCKAR* ↑, *CCR5* ↑, *CRHR1* ↓, *CX3CR1* ↑, *CXCR7* ↓, *DUSP4* ↓, *ELTD1* ↑, *F2R* ↑, *FZD6* ↑, *GPR17* ↓, *GPR83* ↑, *GPR116* ↑, *GPR123* ↓, *GPR126* ↑, *GPR133* ↑, *GPR137* ↓, *GPR153* ↓, *GPR179* ↑, *GPR172B* ↓, *GRM4* ↓, *HRH1* ↑, *HTR2A* ↑, *LPHN3* ↑, *MC4R* ↑, *NTSR1* ↓, *PDE8B* ↑, *PIK3CD* ↓, *PRKCG* ↓, *PTGFR* ↑, *PTK2B* ↓, *RAPGEF3* ↓, *RGR* ↓, *RGS16* ↓, *S1PR5* ↓, *TSHR* ↑	529	0.00314
Circadian Rhythm signalling	*ADCYAP1* ↑, *CRY2* ↓, *GRIN2A* ↓, *GRIN2B* ↓, *GRIN2C* ↓, *PER1* ↓	35	0.00374
Axonal Guidance Signalling	*ABLIM3* ↑, *ADAM11* ↓, *ADAM15* ↓, *ADAM28* ↑, *BCAR1* ↓, *CXCR4* ↑, *EFNA5* ↑, *EPHB6* ↓, *FZD6* ↑, *GIT1* ↓, *GNB1L* ↓, *GNG4* ↑, *GNG7* ↓, *LINGO1* ↓, *MAG* ↓, *PIK3CD* ↓, *PLXNA2* ↓, *PLXNB1* ↓, *PLXNC1* ↑, *PRKCG* ↓, *PRKCH* ↓, *SEMA3E* ↓, *SEMA4C* ↓, *SEMA4D* ↓, *SEMA4F* ↑, *SEMA6B* ↓, *SEMA7A* ↓, *SLIT1* ↑, *SLIT2* ↑, *TUBA8* ↓, *UNC5B* ↓	432	0.00706
Glycerolipid Metabolism	*ALDH1A1* ↓, *ALDH4A1* ↓, *DAGLA* ↓, *DGAT2* ↑, *DGKQ* ↓, *DHRS4* ↓, *GLYCTK* ↓, *LIPE* ↓, *LIPN3* ↓, *MGLL* ↓, *PPAP2C* ↓,	148	0.00798
Pathogenesis of Multiple Schlerosis	*CCR5* ↑, *CXCL9* ↑, *CXCL10* ↑	9	0.00819

## Discussion

Notable variation exists in the tolerance to NEB between individual cows with some animals displaying sensitivity and fail to cycle while other animals remain tolerant and continue to cycle [5]. The aim of this study was to establish the global shifts in gene expression profiles which contribute to animals becoming anovulatory following a period of dietary restriction. Results from the current study are discussed below under the following three comparisons: RA v C, RA v RO, and RO v C. Within the comparisons, the main over-represented pathway functions appear to be immune response (RA v C and RA v RO); energy homeostasis (RA v RO); and prevention of GnRH release (RA v RO).

### Restricted Ovulatory v Control

#### Cellular motility

Of the 6 SDE genes resulting in pathway enrichment for the RO v C comparison, down regulation of 3 of these genes increases cellular motility. *CADH1, DSP* and *TSP4* all exhibited reduced expression in RO relative to C. *CADH1* is a cadherin that encodes a cell-cell adhesion glycoprotein, therefore reduced expression of this gene decreases the strength of cellular adhesion thus increasing cellular motility. *DSP* encodes desmoplakin, a desmosome which occurs at intercellular junctions that tightly link adjacent cells. *TSP4* encodes thrombospondin 4, an adhesive glycoprotein that mediates cell to cell interactions. Cellular motility or neuronal plasticity have previously been up-regulated in rodents when diet was reduced, and it has been suggested that this may contribute to a neuroprotective role in animals undergoing reduced dietary intake, as these processes are important in both learning and memory [23–25]. Another gene with decreased expression in RO relative to C was *PAR-1*. Activation of either PAR-1 or PAR-2 may also have a neuroprotective effect [26, 27], and this is believed to be due to mesotrypsinogen/trypsinogen IV activation. However, no trypsinogen gene displayed increased expression in RO v C. It is therefore possible that there is less of a neuroprotective effect of diet restriction on the RO group. This is further compounded by the fact that neither *IL-1α* nor *IL-1β* were differentially expressed in RO relative to C, indicating that the RO group were not forced to choose between survival and protection of the brain or reproduction.

Thyroid stimulating hormone receptor (*TSHR*) had decreased expression in RO v C. Thyroid stimulating hormone (TSH) binds to TSHR, as does thyroid releasing hormone (TRH) to regulate the secretion of the thyroid hormones through a negative feedback loop. The thyroid hormones (T3 and T4) increase metabolism, growth and proliferation. Stimulation of TSHR can result in increased secretion of thyroid hormones. It is therefore possible that TSHR has reduced expression in RO to prevent the secretion of T3 and T4 which would use up energy that the RO group may not have had. This shows that the RO group were under a certain amount of pressure from the diet restriction but not to the same extent as RA. All animals will become anovulatory eventually under severe long term diet restriction and perhaps reduced expression of TSHR in order to save available energy occurs at the beginning of the process in the shift to anovulation.

### Restricted Anovulatory v Control

#### Immune response

Signals generated by the hypothalamic-pituitary-gonadal (HPG) axis powerfully modulate immune system function [28]. Following pathway analysis the most over-represented pathway within the RA v C comparison was RIG-I-like receptor signalling pathway of which four genes (*CXCL10*, *Q1KLR3*, *IFIH1*, and *IL8)*, all of which have roles in immune function, were up-regulated in RA relative to C. The gene, *CXCL10* encodes a chemokine of the CXC subfamily which binds to CXCR3. Binding of the CXCL10 protein to CXCR3 results in pleiotropic effects, including stimulation of monocytes, natural killer and T-cell migration, and modulation of adhesion molecule expression. It has been suggested that along with the significant role chemokines play in immune response, they may be added to the multiple peptides involved in the regulation of neuroendocrine pathways [29].

Both *CXCL10* and *IL8*, had increased expression in RA relative to C and have proven functions in reducing feed intake in rats [30]. In addition, *IL1α*, another immune functioning gene, had increased expression in RA v C. There are two forms of IL-1, IL-1α and IL-1β, and in most studies their biological activities are indistinguishable [31]. Both versions of IL-1 also bind to the same active receptor, IL-1R [31]. IL-1 has an anorexic effect in rats by stimulating the release of corticotropin releasing factor (CRF) in the hypothalamus [32], which acts to reduce food intake. In our study *CXCL10, IL8* and *IL1α* were not responsible for reduced feed intake as dietary restriction was imposed upon experimental animals. However, the immune system has been shown to possess the ability to block normal reproductive functioning [33]. Both IL-1β and IL-1α have been shown to suppress (through blocking GnRH secretion) and interfere with LH release, respectively [34, 35]. These results show that IL-1 is a strong suppressant of GnRH secretion and therefore reproduction, possibly explaining why an alteration in immune response genes was evident in RA compared to C.

#### Neuroprotection

An alternative reason for these immune genes to be switched on in the hypothalamus following diet restriction is that the energy deficit forces the body to decide between reproduction or immune function. In the tree lizard, albeit quite distant to the cow, when energy reserves are tight there is competition between immune function and reproduction [36]. The most important organ to the body’s survival is the brain and so it is plausible that the immune genes mentioned have increased expression to facilitate a neuroprotective effect. It has been well documented in rodents that diet restriction increases longevity [37], but diet restriction is also known to have a neuroprotective effect [38, 39]. Much of this neuroprotection during diet restriction comes about from neurotrophins including BDNF [40, 41]; NT-3 [41]; NGF and; NT-4/5, which promote the survival of neurons. None of these genes had increased expression in RA v C, however, IL-1 has been shown to stimulate the expression of NGF mRNA [42–45] possibly elucidating a mechanism whereby immune genes, particularly IL-1, are up-regulated in RA to protect the brain. It is very possible that the immune genes are carrying out both functions, preventing secretion of GnRH and performing a neuroprotective role to ensure the brain continues to function as a priority.

Further evidence to the body protecting the brain during diet restriction is the over expression of trypsinogen genes. In the RA v C comparison *TRY2* and *PRSS3* were over expressed. The *PRSS3* gene encodes, due to alternative splicing, both mesotrypsinogen and trypsinogen 4 [46]. It has been proposed that mesotrypsinogen/trypsinogen IV, via activation of Proteinase-activated receptor 1 (PAR-1) or Proteinase-activated receptor 2 (PAR-2), might contribute to neuroprotection in the rat brain [27]. Increased expression of trypsinogen was potentially carrying out a similar function and aiding neuroprotection during diet restriction.

### Restricted Anovulatory v Restricted Ovulatory

#### Energy homeostasis

It is interesting to note that although on the same restricted diet, the comparison between RA and RO returned the largest number of SDE. On initial analysis, there appears to be a considerable response in the hypothalamus to attempt to return the animal to a positive energy balance. *PACA*, also known as *PACAP*, was increased in RA relative to RO. This encodes adenylate cyclase activating polypeptide 1, which acts as a neurotransmitter and neuromodulator. PACA stimulates insulin secretion from the pancreas in mice [47, 48], calves [49] and humans [50]. PACA also stimulates an increase in plasma vasopressin concentrations [51–53]. Vasopressin is key to homeostasis as it regulates water, glucose and salt levels in the blood. Additionally, injection of PACA in the medial basal hypothalamus of ovariectomized ewe has been shown to suppress LH secretion and pulse frequency [54]. This action is presumably through an inhibition of GnRH. Increased PACA in the RA group may be preventing any LH secretion while also increasing the secretion of vasopressin to maximize blood glucose concentrations.

Two genes that potently increase food intake; *AgRP* and *NPY* had increased expression in RA v RO. *AgRP* is almost exclusively expressed in the CNS [55, 56], where its gene product increases food intake as it acts as an antagonist to melanocortin-3 receptor (MC3R) and melanocortin-4 receptor (MC4R) [57, 58]. Activation of MC4R by its agonist α-melanocyte-stimulating hormone (α-MSH), results in decreased food intake [59]. Consistent with this, *MC4R* also had increased expression in RA v RO. Similar results have been observed previously in mice where *AgRP* and *NPY* had increased hypothalamic expression during negative energy balance [60]. AgRP expressing neurons in the hypothalamus have been shown to express NPY [61], indicating a close working relationship between these neuropeptides in the hypothalamus. Recently, Allen et al. [13] showed marked differences in the expression of *NPY* and *AGRP* in the hypothalamus of heifers nutritionally programmed to hasten pubertal onset suggesting that they interact to regulate the reproductive neuroendocrine axis in cattle.

NPY is believed to be the most potent elicitor of food intake [62]. However, the literature provides evidence of both an inhibitory [63] and a stimulatory role [64] on LH secretion in rodents. NPY is believed to exert its effects on LH through GnRH [64–66] which is dose dependant and influenced by stage of cycle. In order for NPY to have an excitatory effect on GnRH release, intermittent hypothalamic NPY receptor activation is required, whereas continuous activation appears to inhibit LH release [67]. In the many studies on ovariectomized mammals, administration of NPY invariably causes an inhibition on GnRH levels [66, 68–71]. Therefore, in order for NPY to have a positive effect on GnRH, suitable ovarian steroid concentrations must exist and NPY receptor activation must be intermittent. If either of these factors are not satisfactory, NPY will suppress GnRH. The increased expression of *NPY* in RA heifers may chronically activate its receptor and therefore inhibit GnRH release. Furthermore, both estradiol and progesterone plasma concentrations were lowest in RA heifers [72] and may have contributed to NPY inhibiting GnRH.

Diet restriction or fasting increases hypothalamic NPY concentrations [71, 73] and mRNA [74] in sheep, and rats [75]. It is therefore surprising that RA had higher *NPY* expression than RO considering both groups were fed the same restricted diet. However, it has been previously observed in rats that peripheral insulin administration suppresses NPY release in the hypothalamic paraventricular nucleus (PVN) [76]. Additionally, central administration of insulin decreases both *NPY* mRNA and NPY concentrations in hypothalamic areas [77]. These findings suggest that the increase in NPY in response to fasting is dependent on low insulin levels. As documented in our previous study [72], using the same animals, insulin and IGF-1 concentrations were higher in RO compared to the two other groups on days −2 and 0. As the feeding phase progressed concentrations of these hormones decreased in both RO and RA and were similar by day 9 and remained stable until slaughter. It is therefore likely that the reason RO heifers do not become anovulatory during diet restriction is that their initially higher insulin, and possibly IGF-1, concentrations prevent an increase in NPY at sufficiently high concentrations to inhibit GnRH. A considerable carry over effect of this was observed. All heifers had two opportunities to ovulate. Within RA four heifers ovulated the 1st dominant follicle (DF) but failed to ovulate the 2nd DF even though insulin and IGF-1 concentrations were lower in RA from the outset. This suggests that it takes some time for metabolic messages, possibly through NPY, to block GnRH in cattle. The prolonged time taken to prevent GnRH release may explain why RO heifers ovulated the 2nd DF even though insulin levels and IGF-1 levels were almost similar to RA heifers at that stage. NPY has been described as one of the essential messenger molecules that serve as a communication bridge between neural processes that regulate reproduction and energy homeostasis [67], and data from this study certainly supports that finding.

Other genes with roles in energy homeostasis had altered expression in the RA v RO comparison. *AGTR1* which encodes type-1 angiotensin II receptor, functioning in salt and thirst desire had decreased expression in RA. *A6QL98* encodes the apelin receptor which is similar to the angiotensin receptor. This receptor had increased expression in RA. Apelin increases water intake in rats following administration [78, 79]. It also has a diuretic effect which may work by decreasing vasopressin levels in the hypothalamus [80]. Apelin is also believed to exert an effect on food intake but its role is contradictory. Taheri et al. [79] found no effect of centrally administered apelin on food intake. However, another study found the exact opposite effect when apelin was centrally administered in rats [81]. One possible reason for this inconsistency is circadian rhythm. It has been observed in rats that i.c.v. injection of apelin during the night has a dose dependent reduction in food intake 2–4 h after injection. Yet, day-time administration of apelin to satiated rats stimulated feeding [82]. If apelin exerts an inhibitory effect on feed intake in cattle, it would be consistent with the theory that RA heifers have increased expression of certain genes in an attempt to stimulate food intake. This is due to the apelin receptor having increased expression perhaps reflecting that there was a decreased requirement for apelin stimulated food inhibition in RA.

Similarly, in our study, *A6QHL2* encoding cholecystokinin-A receptor had increased expression in RA. A cholecystokinin-A receptor knock-out study in mice showed that cholecystokinin diminished food intake by up to 90% through its receptor [83]. The fact that the receptor had increased expression suggests that it was not activated in RA heifers therefore its food inhibition function did not occur. *Q9BGU4* encodes the corticotropin releasing hormone receptor 1 (CRF-R1). This gene had reduced expression in RA v RO. CRF inhibits food intake so possibly the CRF-R is reduced in RA so any CRF produced by stress response cannot fulfil its role in the reduction in food intake but can stimulate adrenocorticotropic hormone (ACTH) secretion. In contrast, *C6KEA7* which encodes corticotropin releasing hormone receptor 2 had increased expression in RA. CRF is primarily involved in the stress response by stimulating the synthesis and release of ACTH from the anterior pituitary gland, which in turn stimulates the release of glucocorticoids from the adrenal gland. Cortisol levels rise during periods of fasting in humans [84], and this is presumably through an increase of CRF in the hypothalamus. Cortisol stimulates gluconeogenesis, particularly in the liver while also inhibiting glucose uptake in muscle and adipose tissue in order to conserve glucose levels. Data from our previous study [72] using the same animals, showed that glucose levels were unaffected by group suggesting a possible reason for this observation. Cortisol also stimulates lipolysis in adipose tissue. Both restricted groups, RA and RO, had elevated beta hydroxybutyrate (BHB) levels compared to C, indicating a greater amount of lipolysis was occurring in these animals. There are therefore reasons for the CRF receptors to have increased expression in RO.

The fact that these genes (*AgRP*, *NPY*, *MC4R*, *A6QL98*, *A6QHL2*, *Q9BGU4*) which are heavily involved in increasing food intake were up-regulated in RA relative to RO even though both groups were on the same restricted individually fed diet reinforces that RA animals are in a greater energy deficit. All animals on a restricted diet will eventually come to a point where the decision must be made to cease reproductive functioning in order to increase the chances of survival. Data shows that RA are forced to make that decision sooner due to lower plasma IGF-1 and insulin levels, which are most probably due to underlying genetic variation in the animals.

#### Immune response

The second observation from the data is that, similar to that observed in RA v C, there was a considerable immune response in RA relative to RO. Genes such as *CXL10, CXCL9, IL-1β,* and *IL-1α* all had increased expression. This again was somewhat surprising as both groups were on the same diet. However, this proves that RA were in a deeper energy deficit than RO and therefore had to up-regulate immune genes for neuroprotection. *IL-1β* and *IL-1α* were in the top 10% of increased fold change genes in RA relative to RO. *TRY2* also had increased expression in RA and as mentioned trypsinogens in the brain are linked with neuroprotection. Due to the GnRH suppressant role of NPY it is more likely that the immune genes *IL-1α* and *IL-1β* do not initially cause anovulation but rather add to the prevention of a re-initiation of the oestrous cycle by GnRH, along with *PACA* and *NTSR1*.

#### Prevention of GnRH secretion

*NTSR1* had reduced expression in RA heifers and this along with other SDE genes *IL1α*, *IL1β*, *NPY* and *PACA*, may act as a method to reduce any LH surge in order to conserve all available energy for biological processes more important to survival. *NTSR1* encodes neurotensin receptor 1. Neurotensin has been implicated in the regulation of GnRH/LH release. In the rat, GnRH neurons co-express mRNA for NTSR1, suggesting that GnRH neurons may be direct targets for activation by neurotensin [85]. It has also been observed that administration of neurotensin directly in the preoptic area of the hypothalamus evokes LH secretion [86, 87]. Furthermore, a blockade of neurotensin signalling reduces the LH surge in the rat [88]. In mice however, neurotensin is thought not to play a direct role in generating the GnRH/LH surge but is regulated by E2 [89].

## Conclusions

**Fig. 1:**
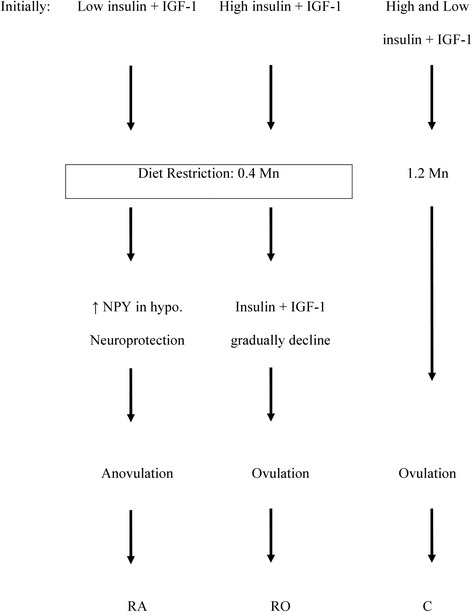
Schematic summary of the effect of diet restriction on heifers

Evidence in this study indicates that heifers became anoestrus following a period of diet restriction. Biological processes affected by dietary restriction in hypothalami included immune response, neuroprotection, cell motility and energy homeostasis. Increased expression of molecules within the hypothalamus provided the GnRH neurons with information that body reserves were not adequate to continue oestrous cyclicity following restriction (Fig 1). There was a delay in this information being sent to the GnRH neurons in RO heifers due to higher initial concentrations of insulin and possibly IGF-1. This was particularly true for *NPY*. The reasoning for RO heifers having higher IGF-1 and insulin concentrations than RA heifers on a similar level of feeding (days −2 and 0) requires more research. However, a possibility is a more efficient utilisation of feed due to underlying genetic variation. The findings presented here for the first time point to a possible molecular mechanism for increased tolerance to an energy deficit in the cow, and assist in the overall understanding of the effects of NEB on fertility in the cow.

## Methods

All animal procedures performed in this study were conducted under experimental licence from the Irish Department of Health and Children (licence number B100/846).in accordance with the Cruelty to Animals Act 1876 and the European Communities (Amendment of Cruelty to Animals Act 1876) Regulation 2002 and 2005. Procedures were carried out in accordance with Regulation 12 of the European Communities Regulations 2006 S.I. 612 of 2006 and were sanctioned by the Research Ethics Committee, University College Dublin (UCD), Ireland.

### Experimental model

A short term (18-day) dietary restriction model was developed which induced abrupt onset of anoestrus in some animals while others maintained oestrous cyclicity [17, 72]. Briefly, in that study 40 Charolais-crossbred heifers exhibiting regular oestrous cycles with an initial liveweight (mean ± SEM) and BCS of 395 ± 3.7 kg, and 2.99 ± 0.04, respectively, were used. Oestrus was synchronised using an 8 day combined CIDR and prostaglandin F_2α_ regimen. During the oestrous synchronisation period all heifers were fed a diet supplying 1.2 estimated maintenance energy requirements (Mn). One day before CIDR removal (day 0), heifers were allocated randomly to either a diet supplying 0.4 Mn (*n* = 28) or retained on 1.2 Mn (C; *n* = 12). Following CIDR removal, ovarian follicular growth and ovulation were monitored using transrectal ultrasonography. On the 11th day after diet allocation, prostaglandin F_2α_ (PGF_2α_) was administered to induce luteolysis, oestrus and ovulation. Within 0.4 Mn, animals were classified as either restricted ovulatory (RO) or restricted anovulatory (RA) depending on whether the DF ovulated or failed to ovulate, respectively. Heifers were blood sampled on days −2, 0–17 and again at slaughter (day 18) for the metabolic hormones IGF-1, insulin, leptin, glucose, BHB, and urea, and the reproductive hormones P4 and E2. After 18 days of feeding all heifers were slaughtered in a commercial abattoir and hypothalamic tissue was recovered. The procedure used for harvesting of hypothalamic tissue has been outlined previously by Matthews [72]. A subset of animals was used for this particular study which consisted of 20 animals (6 C, 7 RO, 7 RA).

### mRNA extraction

Hypothalami were collected, immediately snap frozen in liquid nitrogen and subsequently stored at −80 °C. Total RNA was isolated using a lipid tissue midi kit (Qiagen Ltd., West Sussex, UK) which includes a DNase step to remove any genomic DNA contamination. RNA yield and quality were assessed using automated capillary gel electrophoresis on a Bioanalyzer 2100 with RNA 6000 Nano chips according to manufacturer’s instructions (Agilent Technologies Ireland, Dublin, Ireland). Poly A messenger RNA (mRNA) was purified from 10 μg total RNA using Dynal oligo (dT) magnetic beads (Invitrogen, Bio Sciences ltd., Dublin, Ireland). Oligo(dT) selection was performed twice to ensure minimal carry-over of ribosomal RNA (rRNA).

### cDNA preparation

mRNA was fragmented and then reverse transcribed into cDNA. Zinc mediated fragmentation was performed by adding fragmentation reagent (Ambion, Applied Biosystems, Warrington, UK) to the mRNA and incubation at 70 °C for 5 min. First strand cDNA synthesis was performed using 3 μg of random hexamer primers and SuperScript II (Invitrogen). After the first strand was synthesized, second strand synthesis buffer, dNTPs, RNase H and *E. coli* DNA polymerase I (Invitrogen) were added and incubated for 2.5 h at 16 °C to translate the second-strand synthesis. DNA was then purified using a Qiaquick PCR spin column (Qiagen) and eluted in 30 μl EB buffer (Qiagen).

### Library preparation

The ends of cDNA fragments were repaired with a combination of T4 DNA polymerase (New England BioLabs, ISIS Ltd., Wicklow, Ireland) and *E. coli* DNA polymerase I Klenow fragment (New England BioLabs) which remove 3′-overhangs and fill in 5′-overhangs. A single ‘A’ base was added to the 3′-end of blunt phosphorylated cDNA fragments, using the polymerase activity of Klenow Exo fragment (New England BioLabs), to allow for the ligation of adaptors which have a single 3′-T overhang. The ligated adaptors prepare the cDNA fragments to be hybridized to a flow cell. DNA was purified using gel electrophoresis to allow for templates of uniform length to be sequenced. Seventeen cycles of PCR enrichment was performed on the purified adaptor ligated cDNA templates. Adapter ligated cDNA fragment libraries were run on an Illumina GAII using version 3 sequencing and single read cluster generation kits capable of sequencing 42 bases of each template.

### Read alignment and abundance calculations

RNA-seq reads from each flow cell lane were aligned separately to the *Bos taurus* genome (BCM4 genome assembly) [90] using the ultrafast short read aligner Bowtie version 0.12.5 [91]. Fastq output files from the sequencer were used as input. The following options were specified for bowtie processing: quality scores are ASCII characters equal to the Phred quality scores plus 64 (−-Solexa1.3-quals); the maximum number of mismatches allowed in the first 28 bases is 2 (−n 2, −l 28); suppressing all alignments for any read that had more than 1 reportable alignment (−m 1); retained alignments were reported in SAM format (−S).

Files were sorted according to location in the genome and any read duplicates were deleted in order to normalise for PCR bias. The software package HTseq (version 0.4.4p6) (http://pypi.python.org/pypi/HTSeq) was used to calculate raw counts of transcript coverage for all annotated genes from the ENSEMBL v59 annotation of the bovine genome [92]. The counts for all exons from the samples were collated into one file and any gene with fewer than 5 reads in all samples was excluded from the subsequent statistical analysis of differential gene expression.

### Identification of SDE genes and pathway analysis

Statistical analysis of gene expression was carried out using DEseq (Version 1.1.11) [93] which uses a generalisation of the Poisson model, the negative binomial distribution, to model biological and technical variance and test for differential expression between two experimental conditions. The statistical tests were corrected for multiple testing using the Benjamini and Hochberg (BH) method [94] as implemented in R (version 2.12.0). SDE genes were called at a FDR of 0.1, and these were retained for further analysis. Reads were converted to their human orthologs for gene ontology. Data were normalised for gene length bias and genes were mapped to the Innatedb database [95] for pathway analysis using GOseq [96]. Data were also mapped to the IPA database to gather as much information as possible regarding molecular events occurring in the hypothalamus due to dietary restriction and its effect on reproductive performance in the bovine.

## Additional Files

Additional file 1: Table S1. Description: Differentially expressed annotated genes between restricted anovulatory (RA) and control (C) groups. (DOCX 27 kb)

Additional file 2: Table S2. Description: Differentially expressed annotated genes between restricted ovulatory (RO) and control (C) groups. (DOCX 17 kb)

Additional file 3: Table S3. Description: Differentially expressed annotated genes between restricted anovulatory (RO) and restricted ovulatory (RO) groups. (DOCX 104 kb)

## Abbreviations

ACTH: Adrenocorticotropic hormone
BCS: Body condition score
BH: Benjamini and Hochberg
BHB: Beta hydroxybutyrate
C: Control group
CIDR: Controlled internal drug-releasing device
CRF: Corticotropin releasing factor
DF: Dominant follicle
FDR: False discovery rate
FSH: Follicle stimulating hormone
GEO: Gene expression omnibus
GnRH: Gonadotrophin releasing hormone
GO: Gene ontology
HPG: Hypothalamic-pituitary-gonadal
i.c.v.: Intracerebroventricular
IGF-1: Insulin-like growth factor
IPA: Ingenuity pathway analysis
LH: Luteinizing hormone
MC3R: Melanocortin-3 receptor
MC4R: Melanocortin-4 receptor
Mn: Maintenance energy requirements
NEB: Negative energy balance
NPY: Neuropeptide Y
RA: Restricted anovulatory group
RO: Restricted ovulatory group
SDE: Significantly differentially expressed
TRH: Thyroid releasing hormone
TSH: Thyroid stimulating hormone
TSHR: Thyroid stimulating hormone receptor
α-MSH: α-melanocyte-stimulating hormone
