# Supplementary material for: Effect of short term diet restriction on gene expression in the bovine hypothalamus using next generation RNA sequencing technology
Source: BMC Genomics. 2017 Nov 9;18:857. doi: 10.1186/s12864-017-4265-6 (PMC5680758; doi:10.1186/s12864-017-4265-6)
Supplement: Supplementary file 1 — Description: Differentially expressed annotated genes between restricted anovulatory (RA) and control (C) groups. (DOCX 27 kb) [file 12864_2017_4265_MOESM1_ESM.docx]

**Supplementary Table 1.** Differentially expressed annotated genes between restricted anovulatory (RA) and control (C) groups.

| Symbol | Entrez Gene Name | Log2FoldChange^1^ |
| --- | --- | --- |
| *A6QQP6* | gamma-aminobutyric acid type A receptor gamma1 subunit | 0.39 |
| *ADAM28* | ADAM Metallopeptidase Domain 28 | 2.79 |
| *ADARB2* | adenosine deaminase, RNA specific B2 (inactive) | -0.79 |
| *ADCY1* | adenylate cyclase 1 | -0.43 |
| *ADGRD1* | adhesion G protein-coupled receptor D1 | 0.98 |
| *ADGRL4* | adhesion G protein-coupled receptor L4 | 0.56 |
| *AK8* | adenylate kinase 8 | -0.69 |
| *ANGPT2* | angiopoietin 2 | 0.80 |
| *APOA4* | apolipoprotein A4 | -1.37 |
| *ARFGEF3* | ARFGEF family member 3 | -0.35 |
| *ATF3* | activating transcription factor 3 | 0.70 |
| *ATP8B4* | ATPase phospholipid transporting 8B4 (putative) | 1.48 |
| *BSN* | bassoon presynaptic cytomatrix protein | -0.38 |
| *BTC* | betacellulin | 0.57 |
| *C2* | complement C2 | 0.54 |
| *C4A* | Complement C4A | 0.93 |
| *CALRL* | calcitonin receptor like receptor | 0.44 |
| *CARNS1* | carnosine synthase 1 | -0.39 |
| *CDH23* | cadherin related 23 | -0.60 |
| *CFI* | complement factor I | 0.48 |
| *CHRNA2* | cholinergic receptor nicotinic alpha 2 subunit | -0.38 |
| *CLC* | Charcot-Leyden Crystal Protein | 0.60 |
| *CLEC1A* | C-type lectin domain family 1 member A | 0.95 |
| *CLMN* | calmin | -0.39 |
| *COL15A1* | collagen type XV alpha 1 chain | -1.86 |
| *COL22A1* | collagen type XXII alpha 1 chain | 0.47 |
| *CXCL1* | C-X-C Motif Chemokine Ligand 1 | 0.95 |
| *CXCL10* | C-X-C motif chemokine ligand 10 | 1.06 |
| *CXCL2* | C-X-C motif chemokine ligand 2 | 2.85 |
| *CXCL3* | C-X-C Motif Chemokine Ligand 3 | 1.32 |
| *DAGLA* | diacylglycerol lipase alpha | -0.37 |
| *DAO* | D-amino acid oxidase | -0.82 |
| *DES* | desmin | 0.57 |
| *DLGAP3* | DLG associated protein 3 | -0.34 |
| *DTX3L* | deltex E3 ubiquitin ligase 3L | 0.53 |
| *DYSF* | dysferlin | -0.30 |
| *EMILIN3* | elastin microfibril interfacer 3 | -0.60 |
| *F10* | coagulation factor X | -0.83 |
| *FADS6* | fatty acid desaturase 6 | -0.50 |
| *FAM234A* | family with sequence similarity 234 member A | -0.40 |
| *FAT3* | FAT atypical cadherin 3 | -0.30 |
| *FOLR1* | folate receptor 1 | -1.43 |
| *FOSB* | FosB proto-oncogene, AP-1 transcription factor subunit | 0.58 |
| *GBP2* | guanylate binding protein 2 | 1.18 |
| *GDF10* | growth differentiation factor 10 | -0.46 |
| *GDPD5* | glycerophosphodiester phosphodiesterase domain containing 5 | -0.35 |
| *GJC2* | gap junction protein gamma 2 | -0.41 |
| *GPR17* | G protein-coupled receptor 17 | -0.34 |
| *GRID2IP* | Grid2 interacting protein | -0.71 |
| *GRIK3* | glutamate ionotropic receptor kainate type subunit 3 | -0.46 |
| *GRIN2A* | glutamate ionotropic receptor NMDA typa subunit 2A | -0.63 |
| *GRIN2C* | glutamate ionotropic receptor NMDA type subunit 2C | -0.78 |
| *HAPLN4* | hyaluronan and proteoglycan link protein 4 | -0.76 |
| *HDC* | histidine decarboxylase | 0.39 |
| *HERC6* | HECT and RLD domain containing E3 ubiquitin protein ligase family member 6 | 0.88 |
| *HIP1R* | huntingtin interacting protein 1 related | -0.34 |
| *HSPA6* | heat shock protein family A (Hsp70) member 6 | 0.85 |
| *IFI44L* | interferon induced protein 44 like | 1.22 |
| *IFIH1* | interferon induced with helicase C domain 1 | 0.99 |
| *IFIT1* | interferon induced protein with tetratricopeptide repeats 1 | 1.74 |
| *IFIT2* | interferon induced protein with tetratricopeptide repeats 2 | 2.13 |
| *IFIT3* | interferon induced protein with tetratricopeptide repeats 3 | 1.12 |
| *IGLC1* | Immunoglobulin Lambda Constant 1 | -1.26 |
| *IL1A* | interleukin 1 alpha | 2.46 |
| *IL8* | Interleukin 8 | 2.40 |
| *ITPR1* | inositol 1,4,5-trisphosphate receptor type 1 | -0.31 |
| *KCNC3* | potassium voltage-gated channel subfamily C member 3 | -0.48 |
| *KCNF1* | potassium voltage-gated channel modifier subfamily F member 1 | -0.41 |
| *KCNH6* | potassium voltage-gated channel subfamily H member 6 | -0.70 |
| *KDM4B* | lysine demethylase 4B | -0.33 |
| *LGALS13* | Galectin 13 | 1.06 |
| *LIPE* | lipase E, hormone sensitive type | -0.46 |
| *LIX1* | limb and CNS expressed 1 | 0.64 |
| *LLGL1* | LLGL1, scribble cell polarity complex component | -0.28 |
| *LMOD2* | leiomodin 2 | -1.96 |
| *LTBP1* | latent transforming growth factor beta binding protein 1 | 0.44 |
| *LY6G6E* | lymphocyte antigen 6 family member G6E | -0.65 |
| *MFAP3L* | microfibrillar associated protein 3 like | -0.59 |
| *MGAT5B* | mannosyl (alpha-1,6-)-glycoprotein beta-1,6-N-acetyl-glucosaminyltransferase, isozyme B | -0.40 |
| *MSLN* | mesothelin | -0.44 |
| *MX1* | MX dynamin like GTPase 1 | 0.57 |
| *MX2* | MX dynamin like GTPase 2 | 1.38 |
| *MYH11* | myosin heavy chain 11 | 0.35 |
| *MYO15A* | myosin XVA | 0.74 |
| *MYO1D* | myosin ID | -0.31 |
| *MYRF* | myelin regulatory factor | -0.27 |
| *NDST1* | N-deacetylase and N-sulfotransferase 1 | -0.32 |
| *OAS1* | 2'-5'-Oligoadenylate Synthetase 1 | 1.22 |
| *OAS2* | 2'-5'-oligoadenylate synthetase 2 | 0.98 |
| *ORM1* | orosomucoid 1 | 1.95 |
| *PARP14* | poly(ADP-ribose) polymerase family member 14 | 0.41 |
| *PHLDB1* | pleckstrin homology like domain family B member 1 | -0.23 |
| *PODN* | podocan | -0.38 |
| *PTCHD3* | patched domain containing 3 | -0.72 |
| *PYGM* | glycogen phosphorylase, muscle associated | -0.40 |
| *Q1KLR3/*  *DDX58* | DExD/H-box helicase 58 | 0.69 |
| *RHOBTB1* | Rho related BTB domain containing 1 | -0.35 |
| *RIT1* | Ras like without CAAX 1 | 0.38 |
| *RNASE4* | ribonuclease A family member 4 | 0.65 |
| *RNF213* | Ring Finger Protein 213 | 0.74 |
| *RSAD2* | radical S-adenosyl methionine domain containing 2 | 2.14 |
| *RTP4* | receptor transporter protein 4 | 1.16 |
| *SAMD9* | sterile alpha motif domain containing 9 | 1.00 |
| *SCN4B* | sodium voltage-gated channel beta subunit 4 | -0.34 |
| *SCN7A* | sodium voltage-gated channel alpha subunit 7 | 0.82 |
| *SCNN1B* | sodium channel epithelial 1 beta subunit | -0.78 |
| *SDCCAG3* | serologically defined colon cancer antigen 3 | -0.49 |
| *SEMA7A* | semaphorin 7A (John Milton Hagen blood group) | -0.36 |
| *SLC45A3* | solute carrier family 45 member 3 | -0.59 |
| *SLC6A3* | solute carrier family 6 member 3 | -1.39 |
| *SLFN11* | schlafen family member 11 | 1.53 |
| *SNX29* | sorting nexin 29 | -0.43 |
| *SPATA2* | spermatogenesis associated 2 | -0.41 |
| *SPESP1* | sperm equatorial segment protein 1 | 1.60 |
| *ST3GAL6* | ST3 beta-galactoside alpha-2,3-sialyltransferase 6 | 0.39 |
| *ST8SIA5* | ST8 alpha-N-acetyl-neuraminide alpha-2,8-sialyltransferase 5 | -0.72 |
| *SVEP1* | sushi, von Willebrand factor type A, EGF and pentraxin domain containing 1 | 0.80 |
| *SYNJ2* | synaptojanin 2 | -0.46 |
| *TH* | tyrosine hydroxylase | -0.56 |
| *TMEM176B* | transmembrane protein 176B | 1.07 |
| *TMEM215* | transmembrane protein 215 | 0.55 |
| *TMPRSS9* | transmembrane protease, serine 9 | -1.19 |
| *TRY2* | Trypsinogen 2 | 0.82 |
| *TUBA8* | tubulin alpha 8 | -0.53 |
| *UBA7* | ubiquitin like modifier activating enzyme 7 | 0.77 |
| *UNC5B* | unc-5 netrin receptor B | -0.32 |
| *USP18* | ubiquitin specific peptidase 18 | 1.55 |
| *USP31* | ubiquitin specific peptidase 31 | -0.37 |
| *VWA3A* | von Willebrand factor A domain containing 3A | 0.68 |
| *Wfdc21* | WAP four-disulfide core domain 21 | 1.19 |
| *XAF1* | XIAP associated factor 1 | 0.90 |
| *ZBP1* | Z-DNA binding protein 1 | 2.27 |

^1^ Fold changes are up or down in restricted anovulatory (RA) animals relative to control animals (C).
