# Supplementary material for: Effect of short term diet restriction on gene expression in the bovine hypothalamus using next generation RNA sequencing technology
Source: BMC Genomics. 2017 Nov 9;18:857. doi: 10.1186/s12864-017-4265-6 (PMC5680758; doi:10.1186/s12864-017-4265-6)
Supplement: Supplementary file 2 — Description: Differentially expressed annotated genes between restricted ovulatory (RO) and control (C) groups. (DOCX 17 kb) [file 12864_2017_4265_MOESM2_ESM.docx]

**Supplementary Table 2.** Differentially expressed annotated genes between restricted ovulatory (RO) and control (C) groups.

| Symbol | Entrez Gene Name | log2FoldChange^1^ |
| --- | --- | --- |
| *CA5A* | carbonic anhydrase 5A | 5.48 |
| *CADH1/*  *CDH1* | cadherin 1 | -1.04 |
| *CCDC137* | coiled-coil domain containing 137 | 0.49 |
| *COL15A1* | collagen type XV alpha 1 chain | -1.19 |
| *DSP* | desmoplakin | -0.82 |
| *FMOD* | fibromodulin | -0.58 |
| *FOLR1* | folate receptor 1 | -2.45 |
| *GRID2IP* | Grid2 interacting protein | 0.47 |
| *HBD* | hemoglobin subunit delta | -2.44 |
| *HLA-B* | major histocompatibility complex, class I, B | -0.68 |
| *HLA-E* | Major Histocompatibility Complex, Class I, E | -1.52 |
| *HSPA6* | heat shock protein family A (Hsp70) member 6 | 1.27 |
| *IL34* | interleukin 34 | 0.49 |
| *KLF15* | Kruppel like factor 15 | 0.44 |
| *LDB3* | LIM Domain Binding 3 | 0.89 |
| *PAR1/*  *F2R* | coagulation factor II thrombin receptor | -0.49 |
| *PKD1* | polycystin 1, transient receptor potential channel interacting | 0.33 |
| *PRDM7* | PR/SET Domain 7 | 1.08 |
| *Pzp* | pregnancy zone protein | -1.34 |
| *Q05B55* | Immunoglobulin Kappa Locus | -2.25 |
| *RPPH1* | Ribonuclease P RNA Component H1 | 1.11 |
| *SCIN* | scinderin | -0.63 |
| *SNORA63* | Small Nucleolar RNA, H/ACA Box 63 | 1.45 |
| *SVEP1* | sushi, von Willebrand factor type A, EGF and pentraxin domain containing 1 | 0.93 |
| *TMPRSS9* | transmembrane protease, serine 9 | -0.98 |
| *TSHR* | thyroid stimulating hormone receptor | -1.28 |
| *TSP4* | thrombospondin 4 | -1.43 |
| *ZNF3* | zinc finger protein 3 | -0.51 |

^1^ Fold changes are up or down in restricted ovulatory (RO) animals relative to control animals (C).
