# Supplementary material for: Effect of short term diet restriction on gene expression in the bovine hypothalamus using next generation RNA sequencing technology
Source: BMC Genomics. 2017 Nov 9;18:857. doi: 10.1186/s12864-017-4265-6 (PMC5680758; doi:10.1186/s12864-017-4265-6)
Supplement: Supplementary file 3 — Description: Differentially expressed annotated genes between restricted anovulatory (RO) and restricted ovulatory (RO) groups. (DOCX 104 kb) [file 12864_2017_4265_MOESM3_ESM.docx]

**Supplementary Table 3.** Differentially expressed annotated genes between restricted anovulatory (RO) and restricted ovulatory (RO) groups.

| Symbol | Entrez Gene Name | log2FoldChange^1^ |
| --- | --- | --- |
| *A2M* | alpha-2-macroglobulin | 0.27 |
| *A6QL98* | Apelin Receptor | 0.65 |
| *AARS2* | alanyl-tRNA synthetase 2, mitochondrial | -0.42 |
| *AATK* | apoptosis associated tyrosine kinase | -0.39 |
| *ABCB9* | ATP binding cassette subfamily B member 9 | -0.41 |
| *ABHD17A* | abhydrolase domain containing 17A | -0.47 |
| *ABHD8* | abhydrolase domain containing 8 | -0.32 |
| *ABLIM3* | actin binding LIM protein family member 3 | 0.39 |
| *ABTB1* | ankyrin repeat and BTB domain containing 1 | -0.49 |
| *ABTB2* | ankyrin repeat and BTB domain containing 2 | -0.47 |
| *ACAA1* | acetyl-CoA acyltransferase 1 | -0.32 |
| *ACADS* | acyl-CoA dehydrogenase, C-2 to C-3 short chain | -0.53 |
| *ACAP3* | ArfGAP with coiled-coil, ankyrin repeat and PH domains 3 | -0.25 |
| *ACKR3* | atypical chemokine receptor 3 | -0.39 |
| *ACSL5* | acyl-CoA synthetase long-chain family member 5 | 0.55 |
| *ACTG2* | actin, gamma 2, smooth muscle, enteric | 0.87 |
| *ADAM11* | ADAM metallopeptidase domain 11 | -0.42 |
| *ADAM15* | ADAM metallopeptidase domain 15 | -0.30 |
| *ADAM28* | ADAM Metallopeptidase Domain 28 | 4.58 |
| *ADAMTS4* | ADAM metallopeptidase with thrombospondin type 1 motif 4 | -0.34 |
| *ADAMTSL2* | ADAMTS like 2 | -0.53 |
| *ADAP1* | ArfGAP with dual PH domains 1 | -0.32 |
| *ADARB1* | adenosine deaminase, RNA specific B1 | -0.55 |
| *ADARB2* | adenosine deaminase, RNA specific B2 (inactive) | -1.10 |
| *ADCY1* | adenylate cyclase 1 | -0.66 |
| *ADCYAP1* | Adenylate Cyclase Activating Polypeptide 1 | 0.60 |
| *ADGRL2* | adhesion G protein-coupled receptor L2 | 0.36 |
| *ADGRL3* | adhesion G protein-coupled receptor L3 | 0.31 |
| *ADORA3* | adenosine A3 receptor | 0.31 |
| *AGAP3* | ArfGAP with GTPase domain, ankyrin repeat and PH domain 3 | -0.32 |
| *AGAP4* | ArfGAP With GTPase Domain, Ankyrin Repeat And PH Domain 4 | -0.34 |
| *AGO2* | argonaute 2, RISC catalytic component | -0.50 |
| *AGRP* | agouti related neuropeptide | 0.88 |
| *AGTPBP1* | ATP/GTP binding protein 1 | 0.31 |
| *AGTR1* | type-1 angiotensin II receptor | 0.57 |
| *AHDC1* | AT-hook DNA binding motif containing 1 | -0.39 |
| *AK7* | adenylate kinase 7 | 0.57 |
| *AK9* | adenylate kinase 9 | 1.01 |
| *AKAP1* | A-kinase anchoring protein 1 | -0.95 |
| *ALDH1A1* | aldehyde dehydrogenase 1 family member A1 | -0.41 |
| *ALDH1L1* | aldehyde dehydrogenase 1 family member L1 | -0.55 |
| *ALDH4A1* | aldehyde dehydrogenase 4 family member A1 | -0.56 |
| *ALKBH4* | alkB homolog 4, lysine demethylase | -0.65 |
| *ALLC* | allantoicase | 2.14 |
| *ALPK3* | alpha kinase 3 | -0.56 |
| *ALS2CR12* | amyotrophic lateral sclerosis 2 chromosome region 12 | 1.06 |
| *AMDHD2* | amidohydrolase domain containing 2 | -0.67 |
| *AMIGO2* | adhesion molecule with Ig like domain 2 | 0.42 |
| *AMOTL1* | angiomotin like 1 | -0.34 |
| *ANGPT1* | angiopoietin 1 | 0.47 |
| *ANGPTL2* | angiopoietin like 2 | -0.55 |
| *ANKEF1* | Ankyrin Repeat And EF-Hand Domain Containing 1 | 0.58 |
| *ANKRD34A* | ankyrin repeat domain 34A | -0.49 |
| *ANKRD34C* | ankyrin repeat domain 34C | -0.68 |
| *ANKRD50* | ankyrin repeat domain 50 | 0.37 |
| *ANKRD53* | ankyrin repeat domain 53 | -1.08 |
| *ANKRD55* | ankyrin repeat domain 55 | 0.47 |
| *ANKRD6* | ankyrin repeat domain 6 | 0.41 |
| *ANKRD9* | ankyrin repeat domain 9 | -0.74 |
| *ANKS1A* | ankyrin repeat and sterile alpha motif domain containing 1A | -0.36 |
| *ANKS3* | ankyrin repeat and sterile alpha motif domain containing 3 | -0.42 |
| *ANO6* | anoctamin 6 | 0.49 |
| *ANO8* | anoctamin 8 | -0.33 |
| *ANTXR2* | anthrax toxin receptor 2 | 0.52 |
| *ANXA3* | annexin A3 | 0.40 |
| *ANXA4* | annexin A4 | 0.44 |
| *ANXA7* | annexin A7 | 0.33 |
| *ANXA8* | Annexin A8 | 0.77 |
| *ANXA9* | annexin A9 | -0.34 |
| *APBB1* | amyloid beta precursor protein binding family B member 1 | -0.30 |
| *APOA4* | apolipoprotein A4 | -1.21 |
| *AQP1* | aquaporin 1 (Colton blood group) | 0.75 |
| *ARFGEF3* | ARFGEF family member 3 | -0.32 |
| *ARHGAP23* | Rho GTPase activating protein 23 | -0.33 |
| *ARHGAP33* | Rho GTPase activating protein 33 | -0.43 |
| *ARHGAP39* | Rho GTPase activating protein 39 | -0.41 |
| *ARHGAP44* | Rho GTPase activating protein 44 | -0.26 |
| *ARHGEF1* | Rho guanine nucleotide exchange factor 1 | -0.34 |
| *ARHGEF10* | Rho guanine nucleotide exchange factor 10 | -0.39 |
| *ARHGEF16* | Rho guanine nucleotide exchange factor 16 | -0.67 |
| *ARMC3* | Armadillo Repeat Containing 3 | 0.96 |
| *ARMC6* | armadillo repeat containing 6 | -0.42 |
| *ARRDC2* | arrestin domain containing 2 | -0.63 |
| *ARVCF* | Armadillo Repeat Gene Deleted In Velocardiofacial Syndrome | -0.46 |
| *ASB2* | ankyrin repeat and SOCS box containing 2 | -0.56 |
| *ASB6* | ankyrin repeat and SOCS box containing 6 | -0.40 |
| *ASPSCR1* | ASPSCR1, UBX Domain Containing Tether For SLC2A4 | -0.38 |
| *ATF3* | activating transcription factor 3 | 0.68 |
| *ATG2A* | Autophagy Related 2A | -0.43 |
| *ATG4B* | autophagy related 4B cysteine peptidase | -0.42 |
| *ATP13A2* | ATPase 13A2 | -0.38 |
| *ATP2B2* | ATPase plasma membrane Ca2+ transporting 2 | -0.24 |
| *ATP2B4* | ATPase plasma membrane Ca2+ transporting 4 | 0.28 |
| *ATP5D* | ATP synthase, H+ transporting, mitochondrial F1 complex, delta subunit | -0.29 |
| *ATP8B4* | ATPase phospholipid transporting 8B4 (putative) | 1.43 |
| *ATXN1* | ataxin 1 | -0.57 |
| *AUTS2* | AUTS2, Activator Of Transcription And Developmental Regulator | -0.42 |
| *AXIN1* | axin 1 | -0.52 |
| *B3GAT1* | beta-1,3-glucuronyltransferase 1 | -0.31 |
| *B3GAT3* | beta-1,3-glucuronyltransferase 3 | -0.35 |
| *BAHCC1* | BAH Domain And Coiled-Coil Containing 1 | -0.45 |
| *BCAR1* | BCAR1, Cas family scaffolding protein | -0.62 |
| *BCL2L1* | BCL2 like 1 | -0.25 |
| *BCO2* | beta-carotene oxygenase 2 | 1.16 |
| *BDH2* | 3-hydroxybutyrate dehydrogenase 2 | 0.44 |
| *BICRA* | BRD4 Interacting Chromatin Remodeling Complex Associated Protein | -0.50 |
| *BIN1* | Bridging Integrator 1 | -0.26 |
| *BOK* | BOK, BCL2 family apoptosis regulator | -0.67 |
| *BOP1* | block of proliferation 1 | -0.41 |
| *BRSK1* | BR serine/threonine kinase 1 | -0.31 |
| *BSN* | bassoon presynaptic cytomatrix protein | -0.57 |
| *BST2* | Bone Marrow Stromal Cell Antigen 2 | 0.83 |
| *BTBD11* | BTB domain containing 11 | -0.30 |
| *BTC* | betacellulin | 0.69 |
| *BTG3* | BTG anti-proliferation factor 3 | 0.50 |
| *C15orf59* | chromosome 15 open reading frame 59 | -0.44 |
| *C19orf71* | Chromosome 19 Open Reading Frame 71 | -0.73 |
| *C1orf141* | Chromosome 1 Open Reading Frame 141 | 2.52 |
| *C1orf198* | Chromosome 1 Open Reading Frame 198 | -0.34 |
| *C1S/ Q3SYT3* | complement C1s | 0.66 |
| *C2* | complement C2 | 0.75 |
| *C22orf39* | Chromosome 22 Open Reading Frame 39 | -0.44 |
| *C3AR1/ A4IFF5* | complement C3a receptor 1 | 0.70 |
| *C8orf34* | chromosome 8 open reading frame 34 | 0.79 |
| *C9orf172* | chromosome 9 open reading frame 172 | -0.70 |
| *C9orf43* | chromosome 9 open reading frame 43 | 0.91 |
| *CA11* | carbonic anhydrase 11 | -0.39 |
| *CA7* | carbonic anhydrase 7 | -0.71 |
| *CACFD1* | calcium channel flower domain containing 1 | -0.46 |
| *CACNA2D1* | calcium voltage-gated channel auxiliary subunit alpha2delta 1 | 0.38 |
| *CACNA2D2* | calcium voltage-gated channel auxiliary subunit alpha2delta 2 | 0.34 |
| *CACNG5* | calcium voltage-gated channel auxiliary subunit gamma 5 | -0.64 |
| *CADPS2* | calcium dependent secretion activator 2 | 0.40 |
| *CALCRL/ CALRL* | calcitonin receptor like receptor | 0.33 |
| *CAMK2N2* | calcium/calmodulin dependent protein kinase II inhibitor 2 | -0.31 |
| *CAMSAP1* | calmodulin regulated spectrin associated protein 1 | -0.41 |
| *CAPN10* | calpain 10 | -0.59 |
| *CAPN15* | calpain 15 | -0.54 |
| *CAPN3* | calpain 3 | 0.76 |
| *CAPN3* | Calpain 3 | 0.52 |
| *CARNS1* | carnosine synthase 1 | -0.50 |
| *CASKIN1* | CASK interacting protein 1 | -0.36 |
| *CASKIN2* | CASK interacting protein 2 | -0.37 |
| *CASP4* | caspase 4 | 0.58 |
| *CASP5* | caspase 5 | 0.62 |
| *CASP8/Q2LGB8* | caspase 8 | 0.66 |
| *CASQ2* | calsequestrin 2 | -0.36 |
| *CASTOR2* | cytosolic arginine sensor for mTORC1 subunit 2 | -0.37 |
| *CBLN2/ LOC785870* | cerebellin 2 precursor | -0.44 |
| *CCDC137* | coiled-coil domain containing 137 | -0.37 |
| *CCDC180* | Coiled-Coil Domain Containing 180 | 1.18 |
| *CCDC40* | coiled-coil domain containing 40 | 0.67 |
| *CCDC61* | coiled-coil domain containing 61 | -0.49 |
| *CCDC85B* | Coiled-Coil Domain Containing 85B | -0.62 |
| *CCDC92* | coiled-coil domain containing 92 | -0.29 |
| *CCKAR/ A6QHL2* | cholecystokinin A receptor | 0.55 |
| *CCL2* | C-C motif chemokine ligand 2 | 1.15 |
| *CCM2* | CCM2 scaffolding protein | -0.33 |
| *CCNA1* | cyclin A1 | 1.00 |
| *CCR5* | C-C motif chemokine receptor 5 (gene/pseudogene) | 1.32 |
| *CD14* | CD14 molecule | 0.42 |
| *CD226* | CD226 molecule | 1.06 |
| *CD48* | CD48 molecule | 1.89 |
| *CD53* | CD53 molecule | 0.68 |
| *CD86/ Q1JPC5* | CD86 molecule | 0.51 |
| *CDC42EP1* | CDC42 effector protein 1 | -0.44 |
| *CDC42EP2* | CDC42 effector protein 2 | -0.55 |
| *CDH13* | cadherin 13 | 0.26 |
| *CDH9* | cadherin 9 | 0.54 |
| *CDK18* | cyclin dependent kinase 18 | -0.41 |
| *CDK5R1* | cyclin dependent kinase 5 regulatory subunit 1 | -0.27 |
| *CDK5R2* | cyclin dependent kinase 5 regulatory subunit 2 | -0.64 |
| *Cdkn1c* | cyclin-dependent kinase inhibitor 1C (P57) | -0.50 |
| *CDR2L* | cerebellar degeneration related protein 2 like | -0.59 |
| *CERCAM* | cerebral endothelial cell adhesion molecule | -0.37 |
| *CFAP45* | cilia and flagella associated protein 45 | 0.74 |
| *CFAP52* | Cilia And Flagella Associated Protein 52 | 1.11 |
| *CFAP65* | Cilia And Flagella Associated Protein 65 | 0.74 |
| *CFAP69* | cilia and flagella associated protein 69 | 0.65 |
| *CFAP73* | Cilia And Flagella Associated Protein 73 | 1.42 |
| *CHCHD6* | coiled-coil-helix-coiled-coil-helix domain containing 6 | -0.50 |
| *CHODL* | chondrolectin | 0.58 |
| *CHPF* | chondroitin polymerizing factor | -0.30 |
| *CHRNA2* | cholinergic receptor nicotinic alpha 2 subunit | -0.68 |
| *CHRNA4* | cholinergic receptor nicotinic alpha 4 subunit | -0.59 |
| *CHST2* | carbohydrate sulfotransferase 2 | -0.45 |
| *CIC* | Capicua Transcriptional Repressor | -0.33 |
| *CILP2* | cartilage intermediate layer protein 2 | -1.00 |
| *CIT* | citron rho-interacting serine/threonine kinase | -0.23 |
| *CITED2* | Cbp/p300 interacting transactivator with Glu/Asp rich carboxy-terminal domain 2 | -0.33 |
| *CITED4* | Cbp/p300 interacting transactivator with Glu/Asp rich carboxy-terminal domain 4 | -0.59 |
| *CKAP2L* | cytoskeleton associated protein 2 like | 0.72 |
| *CLASRP* | CLK4 Associating Serine/Arginine Rich Protein | -0.42 |
| *CLDN5* | claudin 5 | -0.40 |
| *CLEC12A* | C-type lectin domain family 12 member A | 0.87 |
| *CLEC1A* | C-type lectin domain family 1 member A | 1.03 |
| *CLEC4F* | C-Type Lectin Domain Family 4 Member F | -0.38 |
| *CLEC7A* | C-type lectin domain containing 7A | 0.93 |
| *CLIC3* | chloride intracellular channel 3 | -0.79 |
| *CLIP2* | CAP-Gly domain containing linker protein 2 | -0.29 |
| *CLMN* | calmin | -0.48 |
| *CLN8* | CLN8, Transmembrane ER And ERGIC Protein | -0.53 |
| *CMYA5* | cardiomyopathy associated 5 | 0.48 |
| *CNTN2* | contactin 2 | -0.41 |
| *COCH* | cochlin | 0.35 |
| *COL15A1* | collagen type XV alpha 1 chain | -0.67 |
| *COL2A1* | collagen type II alpha 1 chain | 0.70 |
| *COL5A2* | Collagen Type V Alpha 2 Chain | 0.49 |
| *COL6A2* | Collagen Type VI Alpha 2 Chain | -0.29 |
| *COL9A2* | collagen type IX alpha 2 chain | -0.49 |
| *COLGALT2* | collagen beta(1-O)galactosyltransferase 2 | 0.42 |
| *COMMD5* | COMM domain containing 5 | -0.43 |
| *COMTD1* | catechol-O-methyltransferase domain containing 1 | -0.74 |
| *CORO7* | Coronin 7 | -0.42 |
| *CPAMD8* | C3 and PZP like, alpha-2-macroglobulin domain containing 8 | -1.75 |
| *CPNE7* | copine 7 | -0.31 |
| *CPNE9* | copine family member 9 | -0.32 |
| *CRHR1/Q9BGU4* | corticotropin releasing hormone receptor 1 | -0.53 |
| *CRHR2/C6KEA7* | corticotropin releasing hormone receptor 2 | 0.78 |
| *CRISPLD2* | cysteine rich secretory protein LCCL domain containing 2 | -0.35 |
| *CRTC1* | CREB regulated transcription coactivator 1 | -0.37 |
| *CRY2* | cryptochrome circadian clock 2 | -0.29 |
| *CRYM* | crystallin mu | 0.39 |
| *CSNK1G2* | casein kinase 1 gamma 2 | -0.33 |
| *CTBP1* | C-terminal binding protein 1 | -0.43 |
| *CUEDC1* | CUE domain containing 1 | -0.32 |
| *CUX2* | cut like homeobox 2 | -0.61 |
| *CX3CR1* | C-X3-C motif chemokine receptor 1 | 0.34 |
| *CXCL10/ CXL10* | C-X-C motif chemokine ligand 10 | 1.88 |
| *CXCL9* | C-X-C motif chemokine ligand 9 | 1.18 |
| *CXCR4* | C-X-C motif chemokine receptor 4 | 0.64 |
| *CXXC7* | CXXC finger protein 7 | -0.41 |
| *CYP24A1* | cytochrome P450 family 24 subfamily A member 1 | 1.02 |
| *CYP27A1* | cytochrome P450 family 27 subfamily A member 1 | -0.30 |
| *CYP2B6* | cytochrome P450 family 2 subfamily B member 6 | 2.12 |
| *CYTIP* | cytohesin 1 interacting protein | 1.16 |
| *DAB2IP* | DAB2 interacting protein | -0.36 |
| *DACT3* | dishevelled binding antagonist of beta catenin 3 | -0.63 |
| *DAGLA* | diacylglycerol lipase alpha | -0.44 |
| *DDX54* | DEAD-box helicase 54 | -0.35 |
| *DDX58* | DExD/H-box helicase 58 | 0.65 |
| *DGAT2* | diacylglycerol O-acyltransferase 2 | 0.46 |
| *DGCR2* | DiGeorge syndrome critical region gene 2 | -0.36 |
| *DGKQ* | diacylglycerol kinase theta | -0.41 |
| *DHRS4* | dehydrogenase/reductase 4 | -0.49 |
| *DIRAS2* | DIRAS family GTPase 2 | -0.48 |
| *DISP2* | dispatched RND transporter family member 2 | -0.32 |
| *DLGAP3* | DLG associated protein 3 | -0.72 |
| *DLL1* | delta like canonical Notch ligand 1 | -0.55 |
| *DMGDH* | dimethylglycine dehydrogenase | 1.68 |
| *DMWD* | dystrophia myotonica, WD repeat containing | -0.40 |
| *DNAH7* | dynein axonemal heavy chain 7 | 0.52 |
| *DNAH9* | dynein axonemal heavy chain 9 | 0.51 |
| *DOT1L* | DOT1 like histone lysine methyltransferase | -0.32 |
| *DR1* | down-regulator of transcription 1 | 0.35 |
| *DRAM1* | DNA damage regulated autophagy modulator 1 | 0.61 |
| *DRC1* | dynein regulatory complex subunit 1 | 0.92 |
| *DSCAML1* | DS cell adhesion molecule like 1 | -0.35 |
| *DTX3L* | deltex E3 ubiquitin ligase 3L | 0.48 |
| *DUSP2* | dual specificity phosphatase 2 | -0.82 |
| *DUSP4* | dual specificity phosphatase 4 | -0.87 |
| *DUSP7* | dual specificity phosphatase 7 | -0.33 |
| *DVL1* | dishevelled segment polarity protein 1 | -0.43 |
| *DYSF* | dysferlin | -0.27 |
| *DZIP3* | DAZ interacting zinc finger protein 3 | 0.33 |
| *E4F1* | E4F transcription factor 1 | -0.41 |
| *ECM2* | extracellular matrix protein 2 | 0.30 |
| *EEF2K* | eukaryotic elongation factor 2 kinase | -0.35 |
| *EEFSEC* | eukaryotic elongation factor, selenocysteine-tRNA specific | -0.46 |
| *EFEMP1* | EGF containing fibulin like extracellular matrix protein 1 | 0.52 |
| *EFNA5* | ephrin A5 | 0.46 |
| *EFS* | embryonal Fyn-associated substrate | -0.37 |
| *EGFL7* | EGF Like Domain Multiple 7 | -0.64 |
| *ELTD1* | adhesion G protein-coupled receptor L4 | 0.70 |
| *EMCN* | endomucin | 0.52 |
| *EMILIN1* | elastin microfibril interfacer 1 | -0.50 |
| *EMILIN3* | elastin microfibril interfacer 3 | -0.83 |
| *EML2* | echinoderm microtubule associated protein like 2 | -0.27 |
| *EML3* | echinoderm microtubule associated protein like 3 | -0.41 |
| *ENGASE* | endo-beta-N-acetylglucosaminidase | -0.41 |
| *ENHO* | Energy Homeostasis Associated | -0.40 |
| *ENO4* | enolase family member 4 | 0.68 |
| *ENPEP* | glutamyl aminopeptidase | 0.46 |
| *ENPP6* | ectonucleotide pyrophosphatase/phosphodiesterase 6 | -0.56 |
| *EOGT* | EGF domain specific O-linked N-acetylglucosamine transferase | 0.39 |
| *EPHB6* | EPH Receptor B6 | -0.33 |
| *EPN1* | epsin 1 | -0.30 |
| *EPN2* | epsin 2 | -0.32 |
| *ERAP2* | endoplasmic reticulum aminopeptidase 2 | 0.55 |
| *ERC2* | ELKS/RAB6-Interacting/CAST Family Member 2 | 0.42 |
| *ESPNL* | espin-like | -0.91 |
| *Esrra* | estrogen related receptor, alpha | -0.66 |
| *EVI2B* | ecotropic viral integration site 2B | 0.57 |
| *EVI5L* | ecotropic viral integration site 5 like | -0.36 |
| *EYA4* | EYA transcriptional coactivator and phosphatase 4 | 0.76 |
| *F10* | coagulation factor X | -0.97 |
| *F2R/PAR1* | coagulation factor II thrombin receptor | 0.34 |
| *F5* | coagulation factor V | 0.64 |
| *FA2H* | fatty acid 2-hydroxylase | -0.49 |
| *FADD* | Fas associated via death domain | -0.76 |
| *FADS6* | fatty acid desaturase 6 | -0.74 |
| *FAM110B* | family with sequence similarity 110 member B | -0.74 |
| *FAM117B* | Family With Sequence Similarity 117 Member B | 0.38 |
| *FAM131B* | family with sequence similarity 131 member B | -0.29 |
| *FAM163A* | family with sequence similarity 163 member A | -0.87 |
| *FAM173A* | family with sequence similarity 173 member A | -0.47 |
| *FAM181B* | family with sequence similarity 181 member B | -0.53 |
| *FAM193A* | family with sequence similarity 193 member A | -0.31 |
| *FAM19A5* | family with sequence similarity 19 member A5, C-C motif chemokine like | -0.43 |
| *FAM20A* | FAM20A, golgi associated secretory pathway pseudokinase | -0.61 |
| *FAM20C* | FAM20C, golgi associated secretory pathway kinase | -0.40 |
| *FAM213B* | family with sequence similarity 213 member B | -0.35 |
| *FAM222A* | family with sequence similarity 222 member A | -0.75 |
| *FAM227A* | family with sequence similarity 227 member A | 0.75 |
| *FAM234A* | family with sequence similarity 234 member A | -0.63 |
| *FAM46A* | family with sequence similarity 46 member A | 0.59 |
| *FAM69B* | family with sequence similarity 69 member B | -0.41 |
| *FAM84A* | family with sequence similarity 84 member A | -0.56 |
| *FAM92B* | family with sequence similarity 92 member B | 0.91 |
| *FAT3* | FAT atypical cadherin 3 | -0.37 |
| *FBRS* | fibrosin | -0.65 |
| *FBRSL1* | fibrosin like 1 | -0.44 |
| *FBXL16* | F-box and leucine rich repeat protein 16 | -0.24 |
| *FBXO2* | F-box protein 2 | -0.34 |
| *FBXO31* | F-box protein 31 | -0.41 |
| *FBXO41* | F-box protein 41 | -0.42 |
| *FBXW5* | F-box and WD repeat domain containing 5 | -0.43 |
| *FCER2* | Fc fragment of IgE receptor II | 2.78 |
| *FCGR2/ FCGR2B* | Fc fragment of IgG receptor IIb | 0.52 |
| *FCGR3* | Fc Fragment Of IgG Receptor IIIa | 0.79 |
| *FCHO1* | FCH domain only 1 | -0.31 |
| *FCRL1* | Fc receptor like 1 | 0.78 |
| *FEM1A* | fem-1 homolog A | -0.31 |
| *FERMT1* | fermitin family member 1 | 0.51 |
| *FEZF1* | FEZ family zinc finger 1 | 0.90 |
| *FGFRL1* | fibroblast growth factor receptor-like 1 | -0.61 |
| *FHOD1* | Formin Homology 2 Domain Containing 1 | -0.39 |
| *FLT1* | fms related tyrosine kinase 1 | 0.30 |
| *FLYWCH1* | FLYWCH-type zinc finger 1 | -0.48 |
| *FMOD* | fibromodulin | 0.74 |
| *Fnbp1l* | formin binding protein 1-like | 0.37 |
| *FNDC3A* | fibronectin type III domain containing 3A | 0.27 |
| *FNDC5* | Fibronectin Type III Domain Containing 5 | -0.45 |
| *FOSB* | FosB proto-oncogene, AP-1 transcription factor subunit | 0.32 |
| *FOXK1* | forkhead box K1 | -0.59 |
| *FSCN1* | fascin actin-bundling protein 1 | -0.36 |
| *FURIN* | furin, paired basic amino acid cleaving enzyme | -0.30 |
| *FYB* | FYN binding protein | 0.53 |
| *FZD6* | frizzled class receptor 6 | 0.44 |
| *GABRG1/ A6QQP6* | gamma-aminobutyric acid type A receptor gamma1 subunit | 0.43 |
| *GABRR2/ GBRR2* | gamma-aminobutyric acid type A receptor rho2 subunit | -0.59 |
| *GADD45B* | growth arrest and DNA damage inducible beta | -0.45 |
| *GAK* | cyclin G associated kinase | -0.30 |
| *GAL3ST3* | galactose-3-O-sulfotransferase 3 | -0.47 |
| *GALNT13* | polypeptide N-acetylgalactosaminyltransferase 13 | 0.39 |
| *GALNT9* | Polypeptide N-Acetylgalactosaminyltransferase 9 | -0.52 |
| *GAS2L1* | growth arrest specific 2 like 1 | -0.46 |
| *GATA3* | GATA binding protein 3 | -0.77 |
| *GATAD2A* | GATA zinc finger domain containing 2A | -0.36 |
| *GBP1* | guanylate binding protein 1 | 1.09 |
| *GBP2* | guanylate binding protein 2 | 0.75 |
| *GDF1* | Growth Differentiation Factor 1 | -0.53 |
| *GDF10* | growth differentiation factor 10 | -0.38 |
| *GDPD5* | glycerophosphodiester phosphodiesterase domain containing 5 | -0.48 |
| *GFRA3* | GDNF family receptor alpha 3 | 1.32 |
| *GHR* | growth hormone receptor | 0.51 |
| *GHRH/ SLIB* | growth hormone releasing hormone | 1.12 |
| *GIPC1* | GIPC PDZ domain containing family member 1 | -0.46 |
| *GIT1* | GIT ArfGAP 1 | -0.39 |
| *GJC2* | gap junction protein gamma 2 | -0.48 |
| *GLB1L* | galactosidase beta 1 like | 0.49 |
| *GLCE* | glucuronic acid epimerase | 0.40 |
| *GLIPR2* | GLI pathogenesis related 2 | 0.55 |
| *GLYCTK* | glycerate kinase | -0.57 |
| *GNB1L* | G protein subunit beta 1 like | -0.61 |
| *GNG4* | G protein subunit gamma 4 | 0.26 |
| *GNG7* | G protein subunit gamma 7 | -0.32 |
| *GPD1* | glycerol-3-phosphate dehydrogenase 1 | -0.25 |
| *GPR116* | adhesion G protein-coupled receptor F5 | 0.32 |
| *GPR123* | adhesion G protein-coupled receptor A1 | -0.54 |
| *GPR126* | adhesion G protein-coupled receptor G6 | 0.58 |
| *GPR133* | adhesion G protein-coupled receptor D1 | 1.04 |
| *GPR137* | G protein-coupled receptor 137 | -0.31 |
| *GPR153* | G protein-coupled receptor 153 | -0.66 |
| *GPR17/ A2VEA2* | G protein-coupled receptor 17 | -0.30 |
| *GPR172B* | G Protein-Coupled Receptor 172B | -0.37 |
| *GPR179* | G protein-coupled receptor 179 | 0.69 |
| *GPR83* | G Protein-Coupled Receptor 83 | 0.70 |
| *GPRIN1* | G protein regulated inducer of neurite outgrowth 1 | 0.38 |
| *GRID2IP* | Grid2 interacting protein | -1.18 |
| *GRIK3* | glutamate ionotropic receptor kainate type subunit 3 | -0.35 |
| *GRIN2A* | Glutamate Ionotropic Receptor NMDA Type Subunit 2A | -0.59 |
| *GRIN2B* | glutamate ionotropic receptor NMDA type subunit 2B | -0.55 |
| *GRIN2C* | glutamate ionotropic receptor NMDA type subunit 2C | -0.95 |
| *GRM4* | glutamate metabotropic receptor 4 | -0.39 |
| *GSN* | gelsolin | -0.35 |
| *GSTO1* | glutathione S-transferase omega 1 | 0.74 |
| *GUCY2C* | guanylate cyclase 2C | 0.64 |
| *H2AFX/Q17QG8* | H2A histone family member X | -0.55 |
| *HAPLN2* | hyaluronan and proteoglycan link protein 2 | -0.45 |
| *HAPLN4* | hyaluronan and proteoglycan link protein 4 | -1.16 |
| *HDAC11* | histone deacetylase 11 | -0.42 |
| *HDAC5* | histone deacetylase 5 | -0.28 |
| *HDC* | histidine decarboxylase | 0.53 |
| *HDHD5* | haloacid dehalogenase like hydrolase domain containing 5 | -0.54 |
| *HERC6* | HECT and RLD domain containing E3 ubiquitin protein ligase family member 6 | 0.49 |
| *HGFAC* | HGF activator | -0.97 |
| *HID1* | HID1 domain containing | -0.27 |
| *HIP1R* | huntingtin interacting protein 1 related | -0.55 |
| *HIPK4* | homeodomain interacting protein kinase 4 | -0.38 |
| *HIRA* | histone cell cycle regulator | -0.35 |
| *HIST4H4* | Histone Cluster 4 H4 | -2.43 |
| *HLA-B* | major histocompatibility complex, class I, B | 0.83 |
| *HLA-DQB1* | major histocompatibility complex, class II, DQ beta 1 | 1.05 |
| *HLA-E* | Major Histocompatibility Complex, Class I, E | 1.41 |
| *HR* | HR, lysine demethylase and nuclear receptor corepressor | -0.35 |
| *HRH1* | histamine receptor H1 | 0.55 |
| *HS6ST1* | heparan sulfate 6-O-sulfotransferase 1 | -0.53 |
| *HSD17B14* | hydroxysteroid 17-beta dehydrogenase 14 | -0.71 |
| *HSPB2* | heat shock protein family B (small) member 2 | -0.89 |
| *HSPG2* | heparan sulfate proteoglycan 2 | -0.44 |
| *HTR2A/ 5HT2A* | 5-hydroxytryptamine receptor 2A | 0.64 |
| *HTT* | huntingtin | -0.25 |
| *HYDIN* | HYDIN, axonemal central pair apparatus protein | 0.84 |
| *ICAM1* | intercellular adhesion molecule 1 | 0.91 |
| *IFI44L* | interferon induced protein 44 like | 0.91 |
| *IFIH1* | interferon induced with helicase C domain 1 | 0.83 |
| *IFIT1* | interferon induced protein with tetratricopeptide repeats 1 | 1.18 |
| *IFIT2* | interferon induced protein with tetratricopeptide repeats 2 | 1.33 |
| *IFIT3* | interferon induced protein with tetratricopeptide repeats 3 | 1.03 |
| *IFRD2* | interferon related developmental regulator 2 | -0.42 |
| *IGFBP5* | insulin like growth factor binding protein 5 | -0.54 |
| *IGFBPL1* | insulin like growth factor binding protein like 1 | 0.54 |
| *IGSF21* | immunoglobin superfamily member 21 | -0.54 |
| *IGSF8* | immunoglobulin superfamily member 8 | -0.42 |
| *IL1A* | interleukin 1 alpha | 2.09 |
| *IL1B* | interleukin 1 beta | 1.32 |
| *IL34* | interleukin 34 | -0.54 |
| *IMPACT* | impact RWD domain protein | 0.37 |
| *IMPDH1* | inosine monophosphate dehydrogenase 1 | -0.30 |
| *INPP5A* | inositol polyphosphate-5-phosphatase A | -0.34 |
| *INPP5J* | Inositol Polyphosphate-5-Phosphatase J | -0.59 |
| *INTS1* | integrator complex subunit 1 | -0.30 |
| *IQGAP1* | IQ Motif Containing GTPase Activating Protein 1 | 0.32 |
| *IQSEC1* | IQ motif and Sec7 domain 1 | -0.35 |
| *IRF2BP1* | interferon regulatory factor 2 binding protein 1 | -0.47 |
| *IRF6* | interferon regulatory factor 6 | -0.49 |
| *IRGQ* | immunity related GTPase Q | -0.48 |
| *IRS2* | insulin receptor substrate 2 | -0.80 |
| *IRX1* | iroquois homeobox 1 | -0.73 |
| *ISL1* | ISL LIM homeobox 1 | 0.59 |
| *ISOC2* | isochorismatase domain containing 2 | -0.54 |
| *ITPK1* | inositol-tetrakisphosphate 1-kinase | -0.42 |
| *JOSD2* | Josephin domain containing 2 | -0.69 |
| *JPH3* | junctophilin 3 | -0.34 |
| *JUP* | junction plakoglobin | -0.33 |
| *KAZN* | kazrin, periplakin interacting protein | -0.38 |
| *KBTBD11* | kelch repeat and BTB domain containing 11 | -0.58 |
| *KCNA1* | potassium voltage-gated channel subfamily A member 1 | -0.64 |
| *KCNA5* | potassium voltage-gated channel subfamily A member 5 | -0.59 |
| *KCNC1* | potassium voltage-gated channel subfamily C member 1 | -0.33 |
| *KCNC3* | potassium voltage-gated channel subfamily C member 3 | -0.69 |
| *KCNF1* | potassium voltage-gated channel modifier subfamily F member 1 | -0.69 |
| *KCNH2* | Potassium Voltage-Gated Channel Subfamily H Member 2 | -0.48 |
| *KCNJ11* | potassium voltage-gated channel subfamily J member 11 | -0.41 |
| *KCNJ12* | potassium voltage-gated channel subfamily J member 12 | -0.57 |
| *KCNJ9* | potassium voltage-gated channel subfamily J member 9 | -0.55 |
| *KCNK12* | potassium two pore domain channel subfamily K member 12 | -0.79 |
| *KCNN1* | potassium calcium-activated channel subfamily N member 1 | -0.77 |
| *KCNQ2* | potassium voltage-gated channel subfamily Q member 2 | -0.29 |
| *KCNQ3* | potassium voltage-gated channel subfamily Q member 3 | -0.38 |
| *KCNS2* | potassium voltage-gated channel modifier subfamily S member 2 | -0.48 |
| *KCNV1* | potassium voltage-gated channel modifier subfamily V member 1 | -0.48 |
| *KCTD12* | potassium channel tetramerization domain containing 12 | -0.37 |
| *KDM4B* | lysine demethylase 4B | -0.43 |
| *KDR* | kinase insert domain receptor | 0.53 |
| *KIAA1324* | KIAA1324 | 0.44 |
| *KIAA1522* | KIAA1522 | -0.45 |
| *KIAA2012* | KIAA2012 | 0.73 |
| *KIAA2013* | KIAA2013 | -0.30 |
| *KIF13B* | kinesin family member 13B | -0.27 |
| *KIR3DX1* | Killer Cell Immunoglobulin Like Receptor, Three Ig Domains X1 | 2.19 |
| *KL* | klotho | 1.30 |
| *KLC2* | kinesin light chain 2 | -0.40 |
| *KLF15* | Kruppel like factor 15 | -0.58 |
| *KLF9* | Kruppel like factor 9 | -0.46 |
| *KLHL1* | kelch like family member 1 | 0.57 |
| *KLHL8* | kelch like family member 8 | 0.47 |
| *KLK6* | kallikrein related peptidase 6 | -0.42 |
| *KNDC1* | Kinase Non-Catalytic C-Lobe Domain Containing 1 | -0.51 |
| *KRT83* | Keratin 83 | -1.25 |
| *L3MBTL3* | l(3)mbt-like 3 (Drosophila) | 0.39 |
| *LAMA5* | laminin subunit alpha 5 | -0.39 |
| *LAMC3* | Laminin Subunit Gamma 3 | -0.49 |
| *LCNL1* | lipocalin like 1 | -0.52 |
| *LCP2* | lymphocyte cytosolic protein 2 | 0.54 |
| *LDB3* | LIM Domain Binding 3 | -1.07 |
| *LENG8* | leukocyte receptor cluster member 8 | -0.31 |
| *LENG9* | leukocyte receptor cluster member 9 | -0.98 |
| *LETM1* | leucine zipper and EF-hand containing transmembrane protein 1 | -0.28 |
| *LGALS13* | Galectin 13 | 1.71 |
| *LIFR* | LIF receptor alpha | -0.32 |
| *LINGO1* | leucine rich repeat and Ig domain containing 1 | -0.37 |
| *LIPE* | lipase E, hormone sensitive type | -0.68 |
| *LIX1* | limb and CNS expressed 1 | 0.80 |
| *LLGL1* | LLGL1, scribble cell polarity complex component | -0.53 |
| *LMF1* | lipase maturation factor 1 | -0.41 |
| *LMNA* | lamin A/C | -0.36 |
| *Lmo3* | LIM domain only 3 | 0.41 |
| *LMOD2* | leiomodin 2 | -1.35 |
| *LMTK2* | lemur tyrosine kinase 2 | -0.45 |
| *LMTK3* | lemur tyrosine kinase 3 | -0.38 |
| *LPIN3* | lipin 3 | -0.63 |
| *LRCH4* | leucine rich repeats and calponin homology domain containing 4 | -0.39 |
| *LRFN3* | leucine rich repeat and fibronectin type III domain containing 3 | -0.51 |
| *LRFN4* | leucine rich repeat and fibronectin type III domain containing 4 | -0.78 |
| *LRIG3* | leucine rich repeats and immunoglobulin like domains 3 | 0.51 |
| *LRRC23* | leucine rich repeat containing 23 | 0.70 |
| *LRRC24* | leucine rich repeat containing 24 | -0.36 |
| *LRRC47* | leucine rich repeat containing 47 | -0.34 |
| *LRRC4B* | leucine rich repeat containing 4B | -0.27 |
| *LRRC61* | leucine rich repeat containing 61 | -0.69 |
| *LRRIQ1* | leucine rich repeats and IQ motif containing 1 | 0.72 |
| *LRRN3* | leucine rich repeat neuronal 3 | 0.34 |
| *LRTM2* | leucine rich repeats and transmembrane domains 2 | -0.99 |
| *LSP1* | lymphocyte-specific protein 1 | -0.61 |
| *LTBP1* | latent transforming growth factor beta binding protein 1 | 0.74 |
| *LY6G6E* | Lymphocyte Antigen 6 Family Member G6E | -0.58 |
| *LZTR1* | leucine zipper like transcription regulator 1 | -0.31 |
| *LZTS2* | leucine zipper tumor suppressor 2 | -0.32 |
| *LZTS3* | leucine zipper tumor suppressor family member 3 | -0.39 |
| *MAG* | myelin associated glycoprotein | -0.22 |
| *MAP1S* | microtubule associated protein 1S | -0.57 |
| *MAP3K11/ A6QQK2* | mitogen-activated protein kinase kinase kinase 11 | -0.46 |
| *MAP3K19* | Mitogen-Activated Protein Kinase Kinase Kinase 19 | 0.65 |
| *MAP3K9* | mitogen-activated protein kinase kinase kinase 9 | -0.34 |
| *MAP7D1* | MAP7 domain containing 1 | -0.33 |
| *MAPK8IP1* | mitogen-activated protein kinase 8 interacting protein 1 | -0.41 |
| *MAPK8IP3* | mitogen-activated protein kinase 8 interacting protein 3 | -0.27 |
| *MAST3* | microtubule associated serine/threonine kinase 3 | -0.33 |
| *MBLAC2* | metallo-beta-lactamase domain containing 2 | 0.33 |
| *MBOAT7* | membrane bound O-acyltransferase domain containing 7 | -0.32 |
| *MC4R* | melanocortin 4 receptor | 0.53 |
| *MCM6* | minichromosome maintenance complex component 6 | 0.38 |
| *MDFIC* | MyoD family inhibitor domain containing | 0.49 |
| *MECOM* | MDS1 and EVI1 complex locus | 0.65 |
| *MED25* | mediator complex subunit 25 | -0.31 |
| *MED7* | Mediator Complex Subunit 7 | 0.51 |
| *METRN* | meteorin, glial cell differentiation regulator | -0.44 |
| *MEX3B* | mex-3 RNA binding family member B | -0.66 |
| *MFAP3L* | microfibrillar associated protein 3 like | -0.81 |
| *MFAP5* | microfibrillar associated protein 5 | 1.86 |
| *MFHAS1* | malignant fibrous histiocytoma amplified sequence 1 | -0.50 |
| *MGAT3* | mannosyl (beta-1,4-)-glycoprotein beta-1,4-N-acetylglucosaminyltransferase | -0.47 |
| *MGAT5B* | mannosyl (alpha-1,6-)-glycoprotein beta-1,6-N-acetyl-glucosaminyltransferase, isozyme B | -0.61 |
| *MGLL* | monoglyceride lipase | -0.49 |
| *MGRN1* | mahogunin ring finger 1 | -0.37 |
| *MIB2* | mindbomb E3 ubiquitin protein ligase 2 | -0.30 |
| *MICALL1* | MICAL like 1 | -0.42 |
| *MIF* | macrophage migration inhibitory factor (glycosylation-inhibiting factor) | -0.38 |
| *MIGA2* | mitoguardin 2 | -0.30 |
| *MKL1* | megakaryoblastic leukemia (translocation) 1 | -0.35 |
| *MMP15* | matrix metallopeptidase 15 | -0.54 |
| *MMRN1* | multimerin 1 | 1.14 |
| *MON1A* | MON1 homolog A, secretory trafficking associated | -0.44 |
| *MPG* | N-methylpurine DNA glycosylase | -0.53 |
| *MPO* | myeloperoxidase | -0.28 |
| *MRPL28* | mitochondrial ribosomal protein L28 | -0.34 |
| *MRPL41* | mitochondrial ribosomal protein L41 | -0.52 |
| *MRPL54* | mitochondrial ribosomal protein L54 | -0.40 |
| *MRPS2* | mitochondrial ribosomal protein S2 | -0.56 |
| *MS4A14* | membrane spanning 4-domains A14 | 1.45 |
| *MSLN* | mesothelin | -0.40 |
| *MSLNL* | Mesothelin-Like | -1.14 |
| *MSR1* | macrophage scavenger receptor 1 | 0.65 |
| *MTSS1L* | MTSS1L, I-BAR domain containing | -0.32 |
| *MUC15* | mucin 15, cell surface associated | 1.06 |
| *MX1* | MX dynamin like GTPase 1 | 0.45 |
| *MX2* | MX dynamin like GTPase 2 | 0.66 |
| *MYADM* | Myeloid Associated Differentiation Marker | 1.32 |
| *MYB* | MYB proto-oncogene, transcription factor | 0.94 |
| *MYH11* | myosin heavy chain 11 | 0.30 |
| *MYH14* | myosin heavy chain 14 | -0.42 |
| *MYO15A* | myosin XVA | 0.61 |
| *MYRF* | myelin regulatory factor | -0.41 |
| *MZF1* | myeloid zinc finger 1 | -0.43 |
| *MZT2B* | mitotic spindle organizing protein 2B | -0.83 |
| *NACC1* | nucleus accumbens associated 1 | -0.40 |
| *NACC2* | NACC family member 2 | -0.61 |
| *NAT14* | N-acetyltransferase 14 (putative) | -0.38 |
| *NAT8L* | N-Acetyltransferase 8 Like | -0.49 |
| *NAXD* | NAD(P)HX dehydratase | -0.46 |
| *NCAPG* | non-SMC condensin I complex subunit G | 0.48 |
| *NCDN* | neurochondrin | -0.22 |
| *NCKAP1L* | NCK associated protein 1 like | 0.54 |
| *NDST1* | N-deacetylase and N-sulfotransferase 1 | -0.31 |
| *NDUFS7* | NADH:ubiquinone oxidoreductase core subunit S7 | -0.38 |
| *NEFH* | neurofilament heavy | -0.24 |
| *NELFA* | Negative Elongation Factor Complex Member A | -0.55 |
| *Nes* | nestin | -0.32 |
| *NEU4* | neuraminidase 4 | -0.80 |
| *NEURL1B* | neuralized E3 ubiquitin protein ligase 1B | -1.02 |
| *NEXN* | nexilin F-actin binding protein | -0.34 |
| *NFE2L3* | nuclear factor, erythroid 2 like 3 | -0.28 |
| *NKG7* | Natural Killer Cell Granule Protein 7 | 0.77 |
| *NKX2-2* | NK2 homeobox 2 | -0.60 |
| *NME3* | NME/NM23 nucleoside diphosphate kinase 3 | -0.60 |
| *NOL4* | nucleolar protein 4 | 0.41 |
| *NOTCH2* | notch 2 | 0.29 |
| *NOV* | nephroblastoma overexpressed | 0.75 |
| *NPC1L1* | NPC1 like intracellular cholesterol transporter 1 | -3.58 |
| *NPPC* | natriuretic peptide C | -0.78 |
| *NPTXR* | neuronal pentraxin receptor | -0.38 |
| *NPVF* | neuropeptide VF precursor | 1.57 |
| *NPY* | neuropeptide Y | 0.88 |
| *NR1H2* | nuclear receptor subfamily 1 group H member 2 | -0.29 |
| *NR5A1* | nuclear receptor subfamily 5 group A member 1 | 1.33 |
| *NRXN2* | neurexin 2 | -0.28 |
| *NT5M* | 5',3'-nucleotidase, mitochondrial | -0.40 |
| *NTN4* | netrin 4 | 0.48 |
| *NTSR1* | neurotensin receptor 1 | -0.74 |
| *NUP205* | nucleoporin 205 | 0.34 |
| *NUP210* | nucleoporin 210 | -0.43 |
| *NUPR1* | nuclear protein 1, transcriptional regulator | 1.11 |
| *NWD1* | NACHT and WD repeat domain containing 1 | 0.32 |
| *NYAP1* | neuronal tyrosine phosphorylated phosphoinositide-3-kinase adaptor 1 | -0.36 |
| *OAS1* | 2'-5'-Oligoadenylate Synthetase 1 | 0.90 |
| *OAS2* | 2'-5'-oligoadenylate synthetase 2 | 0.83 |
| *OCA2* | OCA2 melanosomal transmembrane protein | 0.54 |
| *OCLN* | occludin | 0.39 |
| *OLIG1* | oligodendrocyte transcription factor 1 | -0.53 |
| *OPLAH* | 5-oxoprolinase (ATP-hydrolysing) | -0.41 |
| *OR2W3* | Olfactory Receptor Family 2 Subfamily W Member 3 | -0.47 |
| *ORM1* | orosomucoid 1 | 1.71 |
| *OSBP2* | oxysterol binding protein 2 | -0.45 |
| *OSBPL5* | oxysterol binding protein like 5 | -0.45 |
| *OTOF* | otoferlin | 0.53 |
| *OTP* | orthopedia homeobox | 0.39 |
| *OTUD7A* | OTU deubiquitinase 7A | -0.46 |
| *P3H2* | prolyl 3-hydroxylase 2 | 0.37 |
| *PACA* | adenylate cyclase activating polypeptide 1 | 0.33 |
| *PACSIN1* | protein kinase C and casein kinase substrate in neurons 1 | -0.27 |
| *PACSIN3* | protein kinase C and casein kinase substrate in neurons 3 | -0.49 |
| *PADI2* | Peptidyl Arginine Deiminase 2 | -0.33 |
| *PALLD* | palladin, cytoskeletal associated protein | -0.29 |
| *PAQR9* | progestin and adipoQ receptor family member 9 | -0.65 |
| *PARP12* | poly(ADP-ribose) polymerase family member 12 | 0.48 |
| *PARP9* | poly(ADP-ribose) polymerase family member 9 | 0.45 |
| *PC* | pyruvate carboxylase | -0.32 |
| *PCBP4* | poly(rC) binding protein 4 | -0.30 |
| *PCDH18* | protocadherin 18 | 0.50 |
| *PCSK6* | proprotein convertase subtilisin/kexin type 6 | -0.36 |
| *PDE8B* | phosphodiesterase 8B | 0.35 |
| *PDXP* | pyridoxal phosphatase | -0.28 |
| *PDZD7* | PDZ domain containing 7 | -0.60 |
| *PDZRN4* | PDZ Domain Containing Ring Finger 4 | 0.48 |
| *PELP1* | proline, glutamate and leucine rich protein 1 | -0.38 |
| *PER1* | period circadian clock 1 | -0.38 |
| *PEX6* | peroxisomal biogenesis factor 6 | -0.30 |
| *PGBD5* | piggyBac transposable element derived 5 | -0.36 |
| *PHLDB1* | pleckstrin homology like domain family B member 1 | -0.35 |
| *PHOSPHO1* | phosphoethanolamine/phosphocholine phosphatase | -0.90 |
| *PIGQ* | phosphatidylinositol glycan anchor biosynthesis class Q | -0.40 |
| *PIK3CD* | phosphatidylinositol-4,5-bisphosphate 3-kinase catalytic subunit delta | -0.33 |
| *PIN1* | peptidylprolyl cis/trans isomerase, NIMA-interacting 1 | -0.32 |
| *PINK1* | PTEN induced putative kinase 1 | -0.36 |
| *PIP5K1C* | phosphatidylinositol-4-phosphate 5-kinase type 1 gamma | -0.29 |
| *PITPNM1* | phosphatidylinositol transfer protein membrane associated 1 | -0.27 |
| *PKD1* | polycystin 1, transient receptor potential channel interacting | -0.39 |
| *PKIA* | cAMP-dependent protein kinase inhibitor alpha | 0.31 |
| *PKMYT1* | protein kinase, membrane associated tyrosine/threonine 1 | -0.77 |
| *PLAC8* | Placenta Specific 8 | 1.49 |
| *PLAT* | plasminogen activator, tissue type | -0.28 |
| *PLCH2* | phospholipase C eta 2 | -0.51 |
| *PLEC* | plectin | -0.24 |
| *PLEKHA4* | pleckstrin homology domain containing A4 | 0.69 |
| *PLEKHA6* | Pleckstrin Homology Domain Containing A6 | -0.26 |
| *PLEKHG1* | pleckstrin homology and RhoGEF domain containing G1 | -0.36 |
| *PLIN3* | perilipin 3 | -0.49 |
| *PLPP7* | phospholipid phosphatase 7 (inactive) | -0.48 |
| *PLSCR4* | phospholipid scramblase 4 | 0.42 |
| *PLXNA2* | plexin A2 | -0.34 |
| *PLXNB1/ LOC616798* | plexin B1 | -0.35 |
| *PLXNC1* | plexin C1 | 0.43 |
| *PMFBP1* | polyamine modulated factor 1 binding protein 1 | 1.17 |
| *PNPLA6* | patatin like phospholipase domain containing 6 | -0.30 |
| *PODN* | podocan | -0.35 |
| *PODXL2* | podocalyxin like 2 | -0.45 |
| *POLR3GL* | RNA polymerase III subunit G like | 0.31 |
| *POLRMT* | RNA polymerase mitochondrial | -0.36 |
| *POSTN* | periostin | 0.78 |
| *PPAP2C* | Phosphatidic Acid Phosphatase 2c | -0.50 |
| *PPIL4* | peptidylprolyl isomerase like 4 | 0.34 |
| *PPP1R16A* | protein phosphatase 1 regulatory subunit 16A | -0.42 |
| *PPP1R17* | protein phosphatase 1 regulatory subunit 17 | 0.76 |
| *PPP1R26* | protein phosphatase 1 regulatory subunit 26 | -0.72 |
| *PPP1R32* | protein phosphatase 1 regulatory subunit 32 | 0.79 |
| *PPP1R37* | protein phosphatase 1 regulatory subunit 37 | -0.34 |
| *PRAG1* | PEAK1 related kinase activating pseudokinase 1 | -0.37 |
| *PRAP1* | proline rich acidic protein 1 | -0.92 |
| *PRDM7* | PR/SET Domain 7 | -1.06 |
| *PRKCG* | protein kinase C gamma | -0.45 |
| *PRKCH* | protein kinase C eta | -0.37 |
| *PRMT6* | protein arginine methyltransferase 6 | -0.47 |
| *PRR12* | proline rich 12 | -0.36 |
| *PRR5L* | proline rich 5 like | -0.53 |
| *PRR7* | proline rich 7, synaptic | -0.61 |
| *PRRT3* | proline rich transmembrane protein 3 | -0.55 |
| *PRSS2/TRY2* | protease, serine 2 | 2.00 |
| *PSD2* | pleckstrin and Sec7 domain containing 2 | -0.28 |
| *PTGFR/ PF2R* | prostaglandin F receptor | 0.60 |
| *PTGR1* | prostaglandin reductase 1 | -0.38 |
| *PTGS2* | prostaglandin-endoperoxide synthase 2 | 0.78 |
| *PTK2B* | protein tyrosine kinase 2 beta | -0.28 |
| *PTPN23* | protein tyrosine phosphatase, non-receptor type 23 | -0.28 |
| *PTPN3* | protein tyrosine phosphatase, non-receptor type 3 | -0.39 |
| *PTPRB* | protein tyrosine phosphatase, receptor type B | 0.29 |
| *PTPRC* | protein tyrosine phosphatase, receptor type C | 0.36 |
| *PTPRCAP* | protein tyrosine phosphatase, receptor type C associated protein | -0.65 |
| *PTPRJ* | Protein Tyrosine Phosphatase, Receptor Type J | 0.42 |
| *PTPRN2* | Protein Tyrosine Phosphatase, Receptor Type N2 | -0.30 |
| *PTPRR* | protein tyrosine phosphatase, receptor type R | 0.40 |
| *PTPRS* | protein tyrosine phosphatase, receptor type S | -0.30 |
| *PVALB* | parvalbumin | -0.39 |
| *PVRL2* | Nectin Cell Adhesion Molecule 2 | -0.37 |
| *PYCR3* | pyrroline-5-carboxylate reductase 3 | -0.34 |
| *PYGM* | glycogen phosphorylase, muscle associated | -0.35 |
| *QPCT* | glutaminyl-peptide cyclotransferase | 0.37 |
| *RAB11FIP3* | RAB11 family interacting protein 3 | -0.40 |
| *RAB3IL1* | RAB3A interacting protein like 1 | -0.59 |
| *RABEP2* | rabaptin, RAB GTPase binding effector protein 2 | -0.47 |
| *RABL6* | RAB, member RAS oncogene family like 6 | -0.40 |
| *RAI1* | retinoic acid induced 1 | -0.32 |
| *RAPGEF3* | Rap guanine nucleotide exchange factor 3 | -0.39 |
| *RARRES1* | retinoic acid receptor responder 1 | 0.78 |
| *RASD1* | ras related dexamethasone induced 1 | -0.69 |
| *RASD2* | RASD family member 2 | -0.29 |
| *RASL10B* | RAS like family 10 member B | -0.48 |
| *Rbfox3* | RNA binding protein, fox-1 homolog (C. elegans) 3 | -0.40 |
| *RBM43* | RNA binding motif protein 43 | 0.57 |
| *RELL2* | RELT like 2 | -0.46 |
| *RELN* | reelin | 0.36 |
| *RELT* | RELT, TNF receptor | -0.46 |
| *REPIN1* | replication initiator 1 | -0.36 |
| *RERE* | arginine-glutamic acid dipeptide repeats | -0.33 |
| *REXO1* | RNA exonuclease 1 homolog | -0.44 |
| *RFFL* | ring finger and FYVE like domain containing E3 ubiquitin protein ligase | -0.35 |
| *RGMA* | repulsive guidance molecule family member a | -0.36 |
| *RGR* | retinal G protein coupled receptor | -0.45 |
| *RGS16* | regulator of G protein signaling 16 | -0.52 |
| *RGS22* | regulator of G protein signaling 22 | 1.03 |
| *RGS8* | regulator of G protein signaling 8 | -0.34 |
| *RHOT2* | ras homolog family member T2 | -0.38 |
| *RIMKLA* | ribosomal modification protein rimK like family member A | -0.42 |
| *RMRP* | RNA Component Of Mitochondrial RNA Processing Endoribonuclease | -2.60 |
| *RNASE4* | ribonuclease A family member 4 | 0.46 |
| *RND3* | Rho family GTPase 3 | 0.37 |
| *RNF213* | Ring Finger Protein 213 | 0.55 |
| *ROM1* | retinal outer segment membrane protein 1 | -0.40 |
| *ROPN1* | rhophilin associated tail protein 1 | 1.42 |
| *RORA* | RAR related orphan receptor A | -0.32 |
| *RPPH1* | Ribonuclease P RNA Component H1 | -1.50 |
| *RPS6KB2* | ribosomal protein S6 kinase B2 | -0.51 |
| *RSAD2* | radical S-adenosyl methionine domain containing 2 | 1.16 |
| *RSPH14* | radial spoke head 14 homolog | -0.79 |
| *RSPH4A* | radial spoke head 4 homolog A | 0.93 |
| *RTKN* | rhotekin | -0.34 |
| *RTN4RL1* | reticulon 4 receptor like 1 | -0.60 |
| *RTP4* | receptor transporter protein 4 | 0.81 |
| *RXRG* | retinoid X receptor gamma | 0.64 |
| *S1PR5* | sphingosine-1-phosphate receptor 5 | -0.56 |
| *SAMD9* | sterile alpha motif domain containing 9 | 0.94 |
| *SARDH* | sarcosine dehydrogenase | -0.80 |
| *SART1* | squamous cell carcinoma antigen recognized by T-cells 1 | -0.36 |
| *SCAF1* | SR-related CTD associated factor 1 | -0.47 |
| *SCAND1* | SCAN domain containing 1 | -0.38 |
| *SCARA5* | scavenger receptor class A member 5 | 0.38 |
| *SCARNA12* | Small Cajal Body-Specific RNA 12 | -1.53 |
| *SCIN* | scinderin | 0.59 |
| *SCLY* | selenocysteine lyase | -0.61 |
| *SCN1B* | sodium voltage-gated channel beta subunit 1 | -0.53 |
| *SCN3A* | sodium voltage-gated channel alpha subunit 3 | 0.38 |
| *SCN4B* | sodium voltage-gated channel beta subunit 4 | -0.53 |
| *SCN5A* | sodium voltage-gated channel alpha subunit 5 | 0.48 |
| *SCRIB* | scribbled planar cell polarity protein | -0.41 |
| *SDC3* | syndecan 3 | -0.29 |
| *SDF2L1* | stromal cell derived factor 2 like 1 | -0.56 |
| *SELENOM* | selenoprotein M | -0.60 |
| *SELL* | selectin L | 1.17 |
| *SEMA3E* | semaphorin 3E | -0.36 |
| *SEMA4C* | semaphorin 4C | -0.36 |
| *SEMA4D* | Semaphorin 4D | -0.24 |
| *SEMA4F* | ssemaphorin 4F | 0.41 |
| *SEMA6B* | semaphorin 6B | -0.43 |
| *SEMA7A* | semaphorin 7A (John Milton Hagen blood group) | -0.48 |
| *SFT2D3* | SFT2 Domain Containing 3 | -0.93 |
| *SGPL1* | sphingosine-1-phosphate lyase 1 | 0.30 |
| *SH2B1* | SH2B adaptor protein 1 | -0.38 |
| *SH3BP4* | SH3 domain binding protein 4 | -0.29 |
| *SH3D19* | SH3 domain containing 19 | -0.31 |
| *SH3GLB2* | SH3 domain containing GRB2 like, endophilin B2 | -0.35 |
| *SH3PXD2A* | SH3 and PX domains 2A | -0.55 |
| *SHC2* | SHC adaptor protein 2 | -0.47 |
| *SHF* | Src homology 2 domain containing F | -0.35 |
| *SHOX2* | short stature homeobox 2 | -0.86 |
| *SIMC1* | SUMO interacting motifs containing 1 | 0.39 |
| *SIPA1* | signal-induced proliferation-associated 1 | -0.51 |
| *SIPA1L1* | signal induced proliferation associated 1 like 1 | 0.37 |
| *SKI* | SKI proto-oncogene | -0.45 |
| *SLC11A2* | Solute Carrier Family 11 Member 2 | 0.56 |
| *SLC16A6* | solute carrier family 16 member 6 | -0.51 |
| *SLC1A2* | solute carrier family 1 member 2 | -0.45 |
| *SLC24A2* | solute carrier family 24 member 2 | -0.32 |
| *SLC25A29* | solute carrier family 25 member 29 | -0.37 |
| *SLC27A1* | solute carrier family 27 member 1 | -0.31 |
| *SLC27A2* | solute carrier family 27 member 2 | 0.94 |
| *SLC2A5* | Solute Carrier Family 2 Member 5 | 0.47 |
| *SLC2A6* | solute carrier family 2 member 6 | -0.54 |
| *SLC35B2* | solute carrier family 35 member B2 | -0.32 |
| *SLC35D3* | solute carrier family 35 member D3 | -0.59 |
| *SLC35E4* | Solute Carrier Family 35 Member E4 | -0.70 |
| *SLC35F4* | solute carrier family 35 member F4 | 0.77 |
| *SLC37A2* | solute carrier family 37 member 2 | 0.47 |
| *SLC38A10* | solute carrier family 38 member 10 | -0.40 |
| *SLC39A13* | solute carrier family 39 member 13 | -0.40 |
| *SLC41A3* | Solute Carrier Family 41 Member 3 | -0.56 |
| *SLC45A3* | solute carrier family 45 member 3 | -0.68 |
| *SLC45A4* | solute carrier family 45 member 4 | -0.55 |
| *SLC4A2* | solute carrier family 4 member 2 | -0.42 |
| *SLC6A15* | solute carrier family 6 member 15 | 0.32 |
| *SLC6A3* | Solute Carrier Family 6 Member 3 | -0.81 |
| *SLC6A9* | solute carrier family 6 member 9 | -0.25 |
| *SLC9A3R2* | SLC9A3 Regulator 2 | -0.37 |
| *SLCO2B1* | solute carrier organic anion transporter family member 2B1 | 0.31 |
| *SLFN11* | schlafen family member 11 | 1.59 |
| *SLIT1* | slit guidance ligand 1 | 0.37 |
| *SLIT2* | slit guidance ligand 2 | 0.61 |
| *SMIM5* | Small Integral Membrane Protein 5 | -0.47 |
| *SMPD1* | sphingomyelin phosphodiesterase 1 | -0.30 |
| *SNCB* | synuclein beta | -0.38 |
| *SNORA63* | Small Nucleolar RNA, H/ACA Box 63 | -1.14 |
| *SNPH* | syntaphilin | -0.40 |
| *SNRNP70* | small nuclear ribonucleoprotein U1 subunit 70 | -0.36 |
| *SNX29* | sorting nexin 29 | -0.39 |
| *SOGA1* | suppressor of glucose, autophagy associated 1 | -0.44 |
| *SOGA3* | SOGA Family Member 3 | -0.35 |
| *SORBS3* | sorbin and SH3 domain containing 3 | -0.27 |
| *SORCS2* | Sortilin Related VPS10 Domain Containing Receptor 2 | -0.40 |
| *SOST* | sclerostin | -1.28 |
| *SOX10* | SRY-box 10 | -0.38 |
| *SOX13* | SRY-box 13 | -0.36 |
| *SOX8* | SRY-box 8 | -0.55 |
| *SP100* | SP100 Nuclear Antigen | 0.99 |
| *SPACA9* | sperm acrosome associated 9 | -0.57 |
| *SPAG6* | sperm associated antigen 6 | 0.94 |
| *SPATA13* | spermatogenesis associated 13 | -0.56 |
| *SPATA18* | spermatogenesis associated 18 | 1.09 |
| *SPATA2* | spermatogenesis associated 2 | -0.44 |
| *SPECC1* | sperm antigen with calponin homology and coiled-coil domains 1 | -0.28 |
| *SPEF2* | sperm flagellar 2 | 0.63 |
| *SPHK2* | sphingosine kinase 2 | -0.37 |
| *SPIRE2* | Spire Type Actin Nucleation Factor 2 | -0.32 |
| *SPNS1* | sphingolipid transporter 1 (putative) | -0.35 |
| *SPSB3* | splA/ryanodine receptor domain and SOCS box containing 3 | -0.39 |
| *SPSB4* | splA/ryanodine receptor domain and SOCS box containing 4 | -1.10 |
| *SPTBN4* | spectrin beta, non-erythrocytic 4 | -0.27 |
| *SRCIN1* | SRC kinase signaling inhibitor 1 | -0.42 |
| *SREBF1* | sterol regulatory element binding transcription factor 1 | -0.34 |
| *SSH3* | slingshot protein phosphatase 3 | -0.34 |
| *ST3GAL6* | ST3 beta-galactoside alpha-2,3-sialyltransferase 6 | 0.31 |
| *ST8SIA5* | ST8 alpha-N-acetyl-neuraminide alpha-2,8-sialyltransferase 5 | -0.77 |
| *STAT1/ A4IFU4* | signal transducer and activator of transcription 1 | 0.28 |
| *STEAP4* | STEAP4 metalloreductase | 1.83 |
| *STK32C* | serine/threonine kinase 32C | -0.38 |
| *STUM* | stum, mechanosensory transduction mediator homolog | -0.44 |
| *SULF1* | sulfatase 1 | 0.44 |
| *SUN2* | Sad1 and UNC84 domain containing 2 | -0.27 |
| *SURF6* | surfeit 6 | -0.49 |
| *SYNGR2* | synaptogyrin 2 | -0.35 |
| *SYNJ2* | synaptojanin 2 | -0.52 |
| *SYT3* | synaptotagmin 3 | -0.47 |
| *SYT4* | synaptotagmin 4 | 0.26 |
| *SYT6* | synaptotagmin 6 | 0.57 |
| *TAB1* | TGF-Beta Activated Kinase 1 (MAP3K7) Binding Protein 1 | -0.41 |
| *TAC1/ TKN1* | tachykinin precursor 1 | 0.43 |
| *TACC1* | transforming acidic coiled-coil containing protein 1 | -0.27 |
| *TANGO2* | transport and golgi organization 2 homolog | -0.49 |
| *TAP1* | transporter 1, ATP binding cassette subfamily B member | 0.48 |
| *TBC1D10B* | TBC1 domain family member 10B | -0.29 |
| *TBL3* | transducin beta like 3 | -0.48 |
| *TCF7L2* | Transcription Factor 7 Like 2 | -0.53 |
| *TEKT1* | tektin 1 | 0.83 |
| *TELO2* | telomere maintenance 2 | -0.49 |
| *TEX26* | testis expressed 26 | 1.07 |
| *TFEB* | transcription factor EB | -0.53 |
| *TFPT* | TCF3 fusion partner | -0.55 |
| *TGIF1* | TGFB induced factor homeobox 1 | 0.58 |
| *TGM2* | transglutaminase 2 | 0.39 |
| *THAP3* | THAP domain containing 3 | -0.43 |
| *THBS1* | thrombospondin 1 | 0.47 |
| *THEM6* | thioesterase superfamily member 6 | -0.64 |
| *TIMD4* | T-cell immunoglobulin and mucin domain containing 4 | 2.30 |
| *TK1* | thymidine kinase 1 | -0.51 |
| *TLDC1* | TBC/LysM-associated domain containing 1 | -0.43 |
| *TLL1* | tolloid like 1 | 0.86 |
| *TLL2* | tolloid like 2 | 1.43 |
| *TLN2* | talin 2 | -0.26 |
| *TLR2* | toll like receptor 2 | 0.53 |
| *TLR3* | toll like receptor 3 | 0.61 |
| *TLR4* | toll like receptor 4 | 0.50 |
| *TMC6* | transmembrane channel like 6 | -0.61 |
| *TMCC2* | transmembrane and coiled-coil domain family 2 | -0.45 |
| *TMEM106A* | transmembrane protein 106A | 0.45 |
| *TMEM125* | transmembrane protein 125 | -0.53 |
| *TMEM127* | transmembrane protein 127 | -0.50 |
| *TMEM129* | transmembrane protein 129 | -0.38 |
| *TMEM132A* | transmembrane protein 132A | -0.43 |
| *TMEM141* | transmembrane protein 141 | -0.54 |
| *TMEM151A* | transmembrane protein 151A | -0.40 |
| *TMEM160* | transmembrane protein 160 | -0.69 |
| *TMEM17* | transmembrane protein 17 | 0.47 |
| *TMEM173* | transmembrane protein 173 | 0.50 |
| *TMEM175* | transmembrane protein 175 | -0.29 |
| *TMEM176B* | transmembrane protein 176B | 0.90 |
| *TMEM184B* | transmembrane protein 184B | -0.26 |
| *TMEM189* | transmembrane protein 189 | -0.28 |
| *TMEM215* | transmembrane protein 215 | 0.99 |
| *TNK2* | tyrosine kinase non receptor 2 | -0.33 |
| *TNKS1BP1* | tankyrase 1 binding protein 1 | -0.34 |
| *TNRC18* | trinucleotide repeat containing 18 | -0.38 |
| *TNS2* | tensin 2 | -0.25 |
| *TOR1AIP1* | Torsin 1A Interacting Protein 1 | -0.47 |
| *TP53INP2* | tumor protein p53 inducible nuclear protein 2 | -0.47 |
| *TPGS1* | tubulin polyglutamylase complex subunit 1 | -0.67 |
| *TPRA1* | transmembrane protein adipocyte associated 1 | -0.57 |
| *TPSD1* | Tryptase Delta 1 | -1.37 |
| *TRANK1* | tetratricopeptide repeat and ankyrin repeat containing 1 | 0.28 |
| *TRAPPC12* | Trafficking Protein Particle Complex 12 | -0.36 |
| *TRIM21/ RO52* | tripartite motif containing 21 | 0.56 |
| *TRIM3* | tripartite motif containing 3 | -0.34 |
| *TRIM38* | tripartite motif containing 38 | 0.52 |
| *TRIM6-TRIM34* | TRIM6-TRIM34 Readthrough | 0.61 |
| *TRPC4* | transient receptor potential cation channel subfamily C member 4 | 0.63 |
| *TRPM1* | transient receptor potential cation channel subfamily M member 1 | 0.89 |
| *TSHR* | thyroid stimulating hormone receptor | 1.07 |
| *TSPAN15* | tetraspanin 15 | -0.32 |
| *TTC25* | tetratricopeptide repeat domain 25 | 0.57 |
| *TTYH3* | tweety family member 3 | -0.41 |
| *TUBA8* | tubulin alpha 8 | -0.72 |
| *TUSC5* | tumor suppressor candidate 5 | -1.29 |
| *TYRO3* | TYRO3 protein tyrosine kinase | -0.32 |
| *UBA7* | ubiquitin like modifier activating enzyme 7 | 0.51 |
| *UBAC1* | UBA domain containing 1 | -0.33 |
| *UBALD2* | UBA Like Domain Containing 2 | -0.50 |
| *UBE2M* | ubiquitin conjugating enzyme E2 M | -0.33 |
| *UBE2O* | ubiquitin conjugating enzyme E2 O | -0.26 |
| *UBTD1* | ubiquitin domain containing 1 | -0.62 |
| *UHRF1* | ubiquitin like with PHD and ring finger domains 1 | -0.78 |
| *ULK1* | unc-51 like autophagy activating kinase 1 | -0.38 |
| *UNC13D* | unc-13 homolog D | -0.46 |
| *UNC45A* | unc-45 myosin chaperone A | -0.29 |
| *UNC45B* | unc-45 myosin chaperone B | -1.86 |
| *UNC5B* | unc-5 netrin receptor B | -0.42 |
| *USF2* | upstream transcription factor 2, c-fos interacting | -0.31 |
| *USP18* | ubiquitin specific peptidase 18 | 1.29 |
| *USP31* | ubiquitin specific peptidase 31 | -0.47 |
| *UVSSA* | UV stimulated scaffold protein A | -0.42 |
| *VAMP1* | Vesicle Associated Membrane Protein 1 | -0.24 |
| *VGF* | VGF Nerve Growth Factor Inducible | -0.40 |
| *VGLL4* | vestigial like family member 4 | -0.42 |
| *VPS26B* | VPS26, retromer complex component B | -0.31 |
| *VWA1* | von Willebrand factor A domain containing 1 | -0.47 |
| *VWA3A* | von Willebrand factor A domain containing 3A | 0.75 |
| *VWC2* | von Willebrand factor C domain containing 2 | -0.62 |
| *WASHC1* | WASH complex subunit 1 | -0.34 |
| *WDR66* | WD repeat domain 66 | 0.59 |
| *Wfdc21* | WAP four-disulfide core domain 21 | 1.23 |
| *WIZ* | widely interspaced zinc finger motifs | -0.46 |
| *XAF1* | XIAP associated factor 1 | 1.01 |
| *XRCC3* | X-ray repair cross complementing 3 | -0.48 |
| *XYLT2* | xylosyltransferase 2 | -0.36 |
| *YBX3* | Y-box binding protein 3 | -0.37 |
| *YDJC* | YdjC Chitooligosaccharide Deacetylase Homolog | -0.61 |
| *YJEFN3* | YjeF N-terminal domain containing 3 | -0.68 |
| *ZBP1* | Z-DNA binding protein 1 | 1.48 |
| *ZBTB16* | zinc finger and BTB domain containing 16 | -0.48 |
| *ZBTB17* | zinc finger and BTB domain containing 17 | -0.40 |
| *ZBTB4* | zinc finger and BTB domain containing 4 | -0.29 |
| *ZBTB47* | zinc finger and BTB domain containing 47 | -0.34 |
| *ZBTB7A* | zinc finger and BTB domain containing 7A | -0.54 |
| *ZBTB7B* | zinc finger and BTB domain containing 7B | -0.69 |
| *ZC3HAV1* | zinc finger CCCH-type containing, antiviral 1 | 0.47 |
| *ZDHHC22* | zinc finger DHHC-type containing 22 | -0.34 |
| *ZDHHC5* | zinc finger DHHC-type containing 5 | -0.35 |
| *ZDHHC8* | Zinc Finger DHHC-Type Containing 8 | -0.58 |
| *ZFAND2A* | zinc finger AN1-type containing 2A | -0.47 |
| *ZFHX2* | zinc finger homeobox 2 | -0.39 |
| *ZFP36L2* | ZFP36 ring finger protein like 2 | -0.47 |
| *ZFPM1* | zinc finger protein, FOG family member 1 | -0.67 |
| *ZFPM2* | zinc finger protein, FOG family member 2 | 0.55 |
| *ZFYVE28* | zinc finger FYVE-type containing 28 | -0.54 |
| *ZGPAT* | zinc finger CCCH-type and G-patch domain containing | -0.38 |
| *ZIC1* | Zic family member 1 | -0.68 |
| *ZMIZ2* | zinc finger MIZ-type containing 2 | -0.35 |
| *ZNF184* | zinc finger protein 184 | 0.47 |
| *ZNF205* | zinc finger protein 205 | -0.52 |
| *ZNF3* | zinc finger protein 3 | 0.36 |
| *ZNF316* | Zinc Finger Protein 316 | -0.56 |
| *ZNF335* | zinc finger protein 335 | -0.34 |
| *ZNF385A* | zinc finger protein 385A | -0.53 |
| *ZNF408* | zinc finger protein 408 | -0.57 |
| *ZNF414* | zinc finger protein 414 | -0.43 |
| *ZNF423* | zinc finger protein 423 | -0.31 |
| *ZNF428* | zinc finger protein 428 | -0.35 |
| *ZNF532* | zinc finger protein 532 | -0.28 |
| *ZNF536* | zinc finger protein 536 | -0.53 |
| *ZNF575* | Zinc Finger Protein 575 | -0.52 |
| *ZNF598* | zinc finger protein 598 | -0.53 |
| *ZNF628* | zinc finger protein 628 | -0.79 |
| *ZNF703* | zinc finger protein 703 | -0.58 |
| *ZNF74* | zinc finger protein 74 | -0.55 |
| *ZNF771* | zinc finger protein 771 | -0.42 |
| *ZNF775* | Zinc Finger Protein 775 | -0.67 |
| *ZNF784* | zinc finger protein 784 | -0.49 |
| *ZNF787* | zinc finger protein 787 | -0.61 |
| *ZYX* | zyxin | -0.29 |

^1^ Fold changes are up or down in restricted anovulatory (RA) animals relative to restricted ovulatory animals (RO).
